# Thymosin β4 Regulates Focal Adhesion Formation in Human Melanoma Cells and Affects Their Migration and Invasion

**DOI:** 10.3389/fcell.2019.00304

**Published:** 2019-12-23

**Authors:** Aleksandra Makowiecka, Natalia Malek, Ewa Mazurkiewicz, Ewa Mrówczyńska, Dorota Nowak, Antonina Joanna Mazur

**Affiliations:** Department of Cell Pathology, Faculty of Biotechnology, University of Wrocław, Wrocław, Poland

**Keywords:** melanoma, thymosin β4, focal adhesion, epithelial-mesenchymal transition, invasion, migration

## Abstract

Thymosin β4 (Tβ4), a multifunctional 44-amino acid polypeptide and a member of actin-binding proteins (ABPs), plays an important role in developmental processes and wound healing. In recent years an increasing number of data has been published suggesting Tβ4’s involvement in tumorigenesis. However, Tβ4’s role in melanoma tumor development still remains to be elucidated. In our study we demonstrate that Tβ4 is crucial for melanoma adhesion and invasion. For the purpose of our research we tested melanoma cell lines differing in invasive potential. Moreover, we applied shRNAs to silence *TMSB4X* (gene encoding Tβ4) expression in a cell line with high *TMSB4X* expression. We found out that Tβ4 is not only a component of focal adhesions (FAs) and interacts with several FAs components but also regulates FAs formation. We demonstrate that Tβ4 level has an impact on FAs’ number and morphology. Moreover, manipulation with *TMSB4X* expression resulted in changes in cells’ motility on non-coated and Matrigel^TM^ (resembling basement membrane composition)-coated surfaces and drastically decreased invasion abilities of the cells. Additionally, a correlation between Tβ4 expression level and exhibition of mesenchymal-like [epithelial-mesenchymal transition (EMT)] features was discovered. Cells with lowered *TMSB4X* expression were less EMT-progressed than control cells. Summarizing, obtained results show that Tβ4 by regulating melanoma cells’ adhesion has an impact on motility features and EMT. Our study not only contributes to a better understanding of the processes underlying melanoma cells’ capacity to create metastases but also highlights Tβ4 as a potential target for melanoma management therapy.

## Introduction

The crucial event during melanoma progression is a transition from the superficial spreading stage to invasive propagation to the dermis (Miller and Mihm, 2006). In order to invade, melanoma cells need to acquire the ability to cross the basement membrane through alterations in adhesion and motility properties. During tumorigenesis epithelial cells are transformed into mesenchymal-like cells in a process called the epithelial-mesenchymal transition (EMT). Melanoma cells derive from melanocytes, cells of neural crest origin, which during embryogenesis undergo a process resembling EMT (Kulesa et al., 2013). Melanocytes are tightly attached to the basement membrane and their survival and function depend on interactions with extracellular matrix (ECM) and keratinocytes (Cichorek et al., 2013). The main cell-matrix adhesion structures are focal adhesions (FAs), that are composed of clusters of transmembrane proteins – integrins interacting with their ECM ligands and intracellular multiprotein complexes providing connection to the actin cytoskeleton. These structures are responsible for mechanical anchorage to ECM and extracellular cues transmission into a cell’s interior (Zaidel-Bar et al., 2004).

During tumor progression vast number of cellular properties and processes undergo alteration, which means that along with the adhesive properties, the cells’ motility abilities change. There are two cell movement types described as canonical–mesenchymal, dependent on Rac1 activation, characterized by degradation of ECM and formation of lamellipodia and invadopodia, and ameboidal, dependent on Rho/ROCK1 activity but not on ECM degradation (Krakhmal et al., 2015). Both types of cell’s motility base on dynamic rearrangements of the actin cytoskeleton, which depend on the activity of actin-binding proteins (ABPs), which according to their functions can be divided into several groups. To one of these groups belong proteins sequestering monomeric actin (G-actin) and one example of this set is thymosin β4 (Tβ4) (Shekhar and Carlier, 2017). Several studies showed that Tβ4 affects cells’ migration (reviewed in Goldstein et al., 2005; Sribenja et al., 2013). However, the role of Tβ4 in neoplasia is still unclear. Although overexpression of *TMSB4X* (gene encoding Tβ4) is correlated with patients’ poor prognosis in some types of tumors (Chi et al., 2017), Tβ4 exhibits a suppressive effects in others (Caers et al., 2010). An increasing number of data shows that Tβ4 is involved in EMT and cell differentiation in normal and tumor cells (Ho et al., 2007; Mollinari et al., 2009; Wirsching et al., 2014). It was reported that *in vivo* selected melanoma cell lines expressed Tβ4 at high level (Clark et al., 2000), what was connected with their metastatic potential. However, the role of Tβ4 in melanoma progression has not been thoroughly investigated yet. Therefore in our studies, we decided to unveil the role of Tβ4 in melanoma cells’ motility and EMT progression. We performed experiments on melanoma cells differing in invasion abilities and on cells with lowered expression of *TMSB4X* by application of shRNA. We discovered that Tβ4 level regulates the number and morphology of FAs and probably through that has an impact on adhesion and thus motility of melanoma cells. Moreover, we found out that manipulating with *TMSB4X* expression EMT progression can be influenced.

## Results

### High Tβ4 Expression Is Positively Correlated With Invasiveness of Melanoma Cells

**FIGURE 1:**
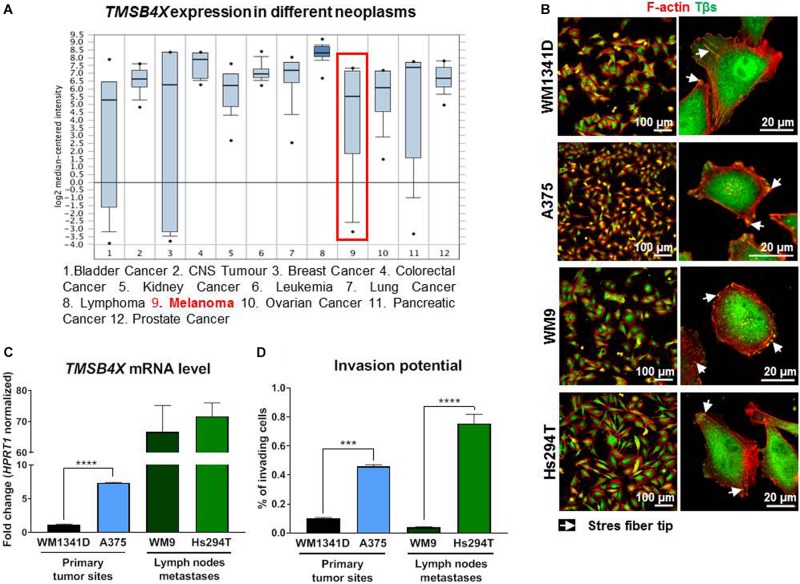
Evaluation of *TMSB4X* expression in different neoplasms and human melanoma cell lines differing in invasion abilities. **(A)** *TMSB4X* expression depending on neoplasia type; adapted from oncomine.org; **(B)** Immunostainings of four human melanoma cell lines to visualize F-actin and Tβs. Left column shows lower magnification, whereas the right one present microphotographs of single cells. **(C)** qRT-PCR analysis of *TMSB4X* expression in cell lines obtained from primary tumor sites and metastases (*n* = 3). **(D)** 3D-migration/invasion analysis of four melanoma cell lines (*n* = 3). Arrows point at stress fibers tips. The significance level was set at ^∗^*P* < 0.05, ^∗∗^*P* < 0.01, ^∗∗∗^*P* < 0.001, and ^∗∗∗∗^*P* < 0.0001 (www.oncomine.org, February 2018, Thermo Fisher Scientific, Ann Arbor, MI, United States).

According to Oncomine database^1^ (Figure 1A; Ramaswamy et al., 2001) the *TMSB4X* expression level varies depending on tumor type. Some of them, including melanoma, are characterized by a very wide range of expression level in patients’ samples. Intrigued by this finding we decided to test four melanoma cell lines in terms of Tβ4 level and its subcellular localization. Here we have to state that validation of two commercially available antibodies recognizing Tβ4 revealed their non-specificity, as two homologous polypeptides to Tβ4 present in humans: Tβ10 and Tβ15 (Goldstein et al., 2005) were recognized by used antibodies (Supplementary Figure S1). We cloned all three thymosins β (Tβs) under a HA-tag and after transfection of the cells with DNA constructs coding for HA-Tβs we fixed and immunostained the cells with antibodies. As it can be seen on micrographs all three Tβs are recognized by two used commercially available antibodies directed against Tβ4. Because of that, starting from now on, whenever antibodies recognizing Tβs are used, we write Tβs instead of Tβ4. Due to the lack of specific antibodies it was also impossible to perform Western blot analysis to verify the level of Tβ4 in studied cells. That is why we checked *TMSB4X* expression level at mRNA level. Analysis of amplification curves (qRT-PCR) showed that among three Tβs present in human in WM1341D cells Tβ4 is a dominant version of Tβs, although Tβ15 is expressed at a relatively high level too (Supplementary Figure S2). In the case of A375 cells the differences between amplification curves for Tβ4 and Tβ15 are much bigger in comparison to WM1341D cell line. On contrary in both cell lines *TMSB10* was expressed at a very low level.

We then examined Tβs subcellular localization. In tested cell lines thymosins β were localized in cell nucleus, cytoplasm and on the cell’s perimeter and they colocalized with filamentous actin (F-actin) at the tips of stress fibers (Figure 1B). Next we found out that metastatic cell lines expressed *TMSB4X* at higher level when compared to cell lines isolated from primary tumor sites (Figure 1C). Interestingly in every pair (primary sites and metastases) there was a cell line with significantly lower *TMSB4X* expression. These observations correlated with invasion capacities of tested cells. The invasion assay was performed using Transwell^TM^ filters coated with Matrigel^TM^ (a mixture of ECM proteins which mimic the basement membrane composition). This assay imitates penetration of the basement membrane by cells during melanoma progression from RPG to VPG. The cells with invasion potential are capable to protrude through Matrigel^TM^ and overcome the pores in Transwell filter’s membrane. The percent of invading A375 cells was three times higher comparing to WM1341D cells, whereas among Hs294T cells there were seven times more invading cells than in the case of WM9 cells (Figure 1D). For further experiments we chose WM1341D and A375 cell lines because they represent cells isolated from primary tumor sites.

### Tβ4 Is a Component of Focal Adhesions and Interacts With FAs Components

**FIGURE 2:**
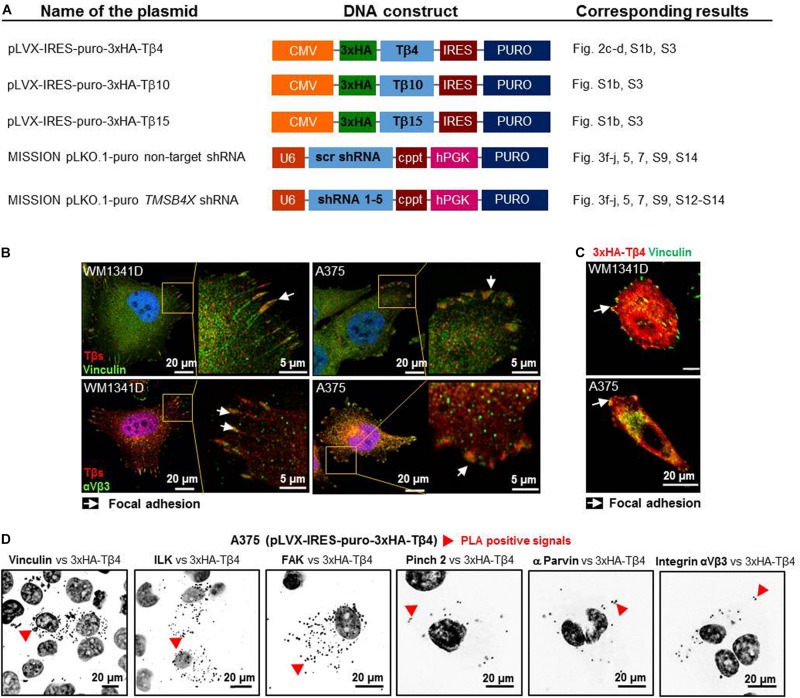
Thymosin β4 is a constituent of focal adhesions (FAs) and forms complexes with FAs constituents in WM1341D and A375 cell lines. **(A)** Schematic representation of DNA constructs used in this study. **(B)** Immunocytochemical stainings visualizing FAs components: vinculin and αVβ3 integrin and co-localizing with them Tβs. **(C)** Visualization of vinculin and exogenous HA-Tβ4 in transfected cells. **(D)** PLA of HA-Tβ4 vs. vinculin, ILK, FAK, Pinch 2, α parvin, and αVβ3 integrin in transfected cells. Cell nuclei are shown in dark gray. Arrows point at FAs, while red arrowheads indicate PLA complexes.

Due to the lack of specific Tβ4-recognizing antibodies we obtained DNA constructs (Figure 2A), which were used in following experiments. Intrigued by Tβs presence at the tips of stress fibers (Figure 1C) we performed additional immunostainings on WM1341D and A375 cells and stated that Tβs co-localized within FAs with vinculin and αVβ3 integrin (FAs’ constituents; Horzum et al., 2014) in studied cell lines (Figure 2B). Further analysis revealed that HA-Tβ4 co-localized with vinculin in FAs (Figure 2C). Moreover, we noted that only after overexpression of Tβ4, but not after Tβ10 or Tβ15 overexpression morphology of FAs was changed (Supplementary Figure S3). In both cell lines FAs were bigger than in non-transfected cells. Proximity ligation assay (PLA) with FAs’ structural and signaling proteins showed that HA-Tβ4 formed complexes with vinculin, integrin-linked (ILK), focal adhesion kinase (FAK), pinch 2, α parvin, and integrin αVβ3 (Figure 2D). Interestingly, HA-Tβ4 is in close proximity to vinculin, ILK and FAK in the whole cell body, whereas putative complexes between HA-Tβ4 and pinch 2, α parvin and integrin αVβ3 are localized rather submembranous, possibly within FAs. Negative controls for PLA assay are shown in Supplementary Figure S4A. All tested here proteins are present at FAs in both cell lines (Supplementary Figure S4B).

### Tβ4 Influences Formation of FAs and Adhesion of Melanoma Cells

**FIGURE 3:**
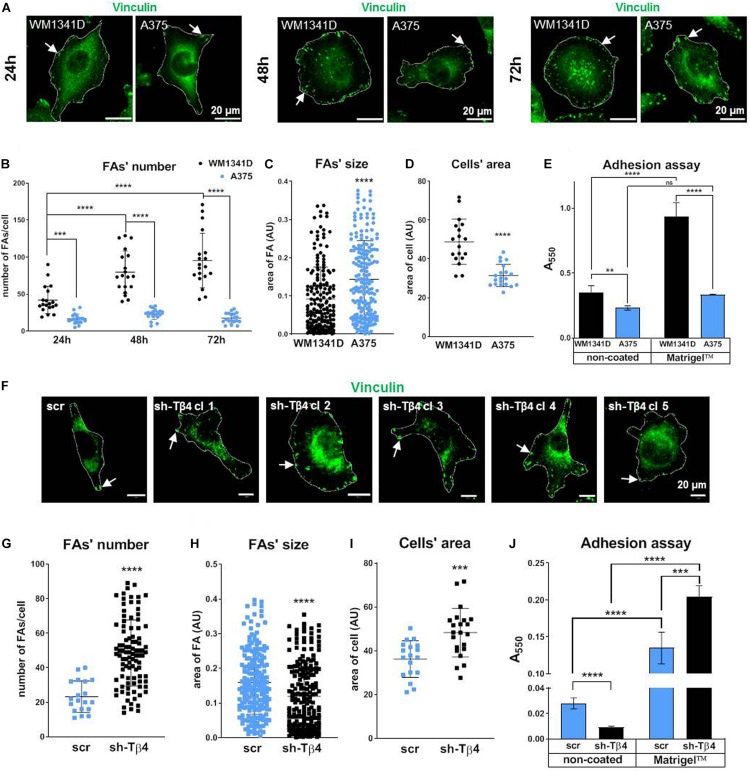
Focal adhesion formation in WM1341D and A375 cells and effect of *TMSB4X* expression silencing on formation of FAs in melanoma cells. **(A,F)** Immunocytochemical analysis of cells in order to visualize vinculin. Cells’ borders were marked for better orientation with white lines. Quantitation of FAs’ number **(B,G)**, size of FA’s **(C,H),** and cell’s area **(D,I)** (*n* = 20). **(E,J)** Adhesion assays of investigated cell lines and clones (*n* = 3). Arrows point at FAs. The significance level was set at ^∗^*P* < 0.05, ^∗∗^*P* < 0.01, ^∗∗∗^*P* < 0.001, and ^∗∗∗∗^*P* < 0.0001 (www.oncomine.org, February 2018, Thermo Fisher Scientific, Ann Arbor, MI, United States).

Quantitative analysis of FAs visualized by vinculin staining (Figure 3A) revealed significantly reduced number (two times less) of FAs in A375 cells when compared to WM1341D cells at 24 h (Figure 3B). At 24, 48, and 72 h average FAs number in WM1341D cells was 43, 80, 95, respectively, meaning that new FAs were additionally formed over time. However, in A375 cells at every time point the FAs number was the same c.a. 20 (Figures 3A,B). Though the number of FAs in WM1341D cells was high, FAs were significantly smaller than those observed in A375 cells (Figures 3A,C). The differences in FAs’ morphology were not caused by changes in vinculin protein level (Supplementary Figure S5). We performed similar analyses on cells with visualized α parvin to check if data obtained on the basis of vinculin staining was replicable (Supplementary Figure S6). Results acquired by analysis of the cells with visualized α parvin corroborated analogous previous outcomes. Next we looked at the area covered by a single cell. On average A375 cell’s area was about 35% smaller than of WM1341D cell (Figure 3D), but in detached cells the differences in median of relative size (FSC parameter measured using flow cytometry) between two cell lines was below 2.5% (Supplementary Figure S7). Therefore the differences observed in cell’s area of A375 and WM1341D cells were caused by distinct adhesion abilities of these cell lines. Because of that, we decided to perform an adhesion assay. Higher number of WM1341D cells adhered within 1 h to both non-coated and Matrigel^TM^-coated surface in comparison to A375 cells (Figure 3E). Intriguingly, while more WM1341D cells attached to Matrigel^TM^ than to non-coated surface, A375 cells adhered to both types of surfaces at the same extent.

To check the role of Tβ4 in FAs formation we obtained five A375 stable clones with decreased *TMSB4X* expression (Supplementary Figure S8). We noted that *TMSB4X* silencing did not affect *TMSB10* and *TMSB15* expression at the mRNA levels (Supplementary Figure S8). Due to unspecifity of antibodies we were not able to observe changes at protein level using immunocytochemical analysis of A375-sh*TMSB4X* and A375-shscramble clones with anti-Tβ4 antibodies (Supplementary Figure S1C). We performed all following analyses for five biological replicates of A375-sh*TMSB4X* clones and then we merged obtained data and described them as shTβ4 on the graphs to simplify the results presentation. First we immunostained the cells in order to visualize vinculin and thus FAs. In A375-sh*TMSB4X* clones we noted higher number but decreased size of FAs when compared to A375-shscramble cells (Figures 3F–H). Again we did analogous analyses on micrographs of cells stained for α parvin and we obtained similar results (Supplementary Figure S9) as we did on the basis of vinculin visualization. Simultaneously, we observed a cell’s area increased by about 33% in A375-sh*TMSB4X* clones (Figure 3I). Next, we checked adhesion abilities of tested clones. Unexpectedly, we noted that cells with lowered *TMSB4X* expression adhered to the non-coated surface in lower numbers than the control cells (Figure 3J). We suspect that the difference between cell adhesion vs. spreading on the surface may be the result of the duration of experiments. In the case of adhesion studies, the cells’ binding was checked after 1 h, while the spreading on the surface after 24 h. On the other hand, cells with lowered level of Tβ4 attached to the Matrigel^TM^-coated surface better than control cells. Generally, both cell types adhered better to Matrigel^TM^ than to non-coated surface. We planned to obtain stable clones from WM1341D cells with increased expression of Tβ4. Unfortunately, we were not able to receive these clones. WM1341D cells turned out to be highly resistant for any kind of genetic manipulation. Transfection efficiency was too low to perform any experiments after transient transfection. We tried lipofection, magnetic transfection and electroporation. We were able to obtain very few transfected cells enabling us to prepare Supplementary Figure S3. However, any attempt to obtain stable clones failed.

### Tβ4 Affects Melanoma Cells’ Invasion and Migration Abilities

**FIGURE 4:**
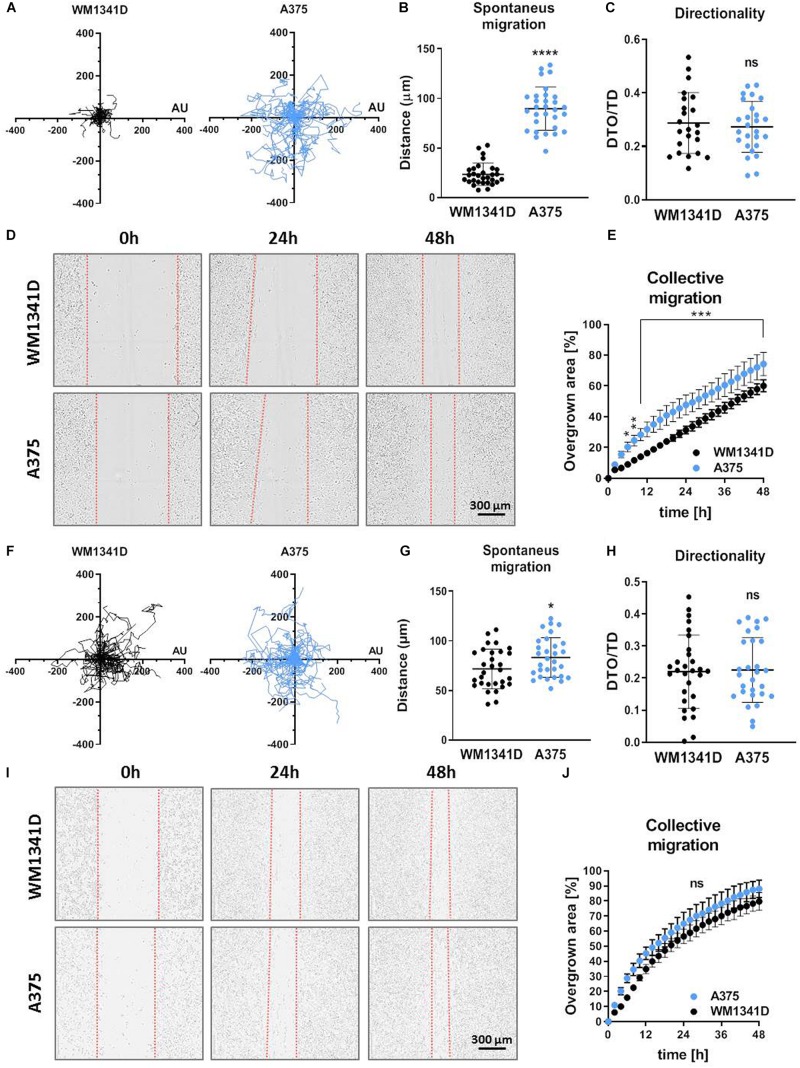
Characterization of 2D motility on non-coated and Matrigel^TM^-coated surface of WM1341D and A375 cells. The cells were seeded either on non-coated surface **(A–E)** or on Matrigel^TM^-coated surface **(F–J)**. **(A–C,F–H)** Spontaneous 2D-migration of tested cells. The cells were recorded over 72 h (*n* = 30): **(A,F)** trajectories of single cells migration, **(B,G)** calculated migrated distances of the cells, **(C,H)** estimation of directionality of cells’ migration. Wound healing assay performed on tested cells (*n* = 3): **(D,I)** representative pictures of cells migrating collectively and **(E,J)** graphs representing scratch closure expressed in % of closed scratch. The significance level was set at ^∗^*P* < 0.05, ^∗∗^*P* < 0.01, ^∗∗∗^*P* < 0.001, and ^∗∗∗∗^*P* < 0.0001 (www.oncomine.org, February 2018, Thermo Fisher Scientific, Ann Arbor, MI, United States).

Because adhesion is directly connected to motility (Huttenlocher et al., 1995), we evaluated migratory abilities of studied cells. Spontaneous 2D cell movement (2D migration) showed that A375 cells covered significantly longer distances (twofold change) compared to WM1341D cells (Figures 4A,B). However, there were no differences between two cell lines in terms of directionality (Figure 4C). The way we calculated this parameter is presented in Supplementary Figure S10. A similar observation was made for collective migration capacity measured by a wound healing assay. The scratch was closed significantly faster by A375 cells when compared to WM1341D cells (Figures 4D,E). We also checked 2D migration of tested cells on Matrigel^TM^-coated surface (Figures 4F–J). In the case of scratch assay we checked first if Matrigel^TM^ coating remained upon making a scratch. Visualization of laminin (main component of Matrigel^TM^) revealed that the layer of Matrigel^TM^ stays after scratching (Supplementary Figure S11). Surprisingly discrepancies between WM1341D and A375 cells were much smaller than in the case of non-coated surface (Figures 4F–J vs. 4A–E). The only statistically significant difference we noted was in the amount of covered distance. A375 cells covered c.a. 1.2 times longer distance than WM1341D cells, whilst on non-coated surface A375 cells moved over c.a. 3.8 times longer distances when compared to WM1341D cells (Figures 4B,G).

**FIGURE 5:**
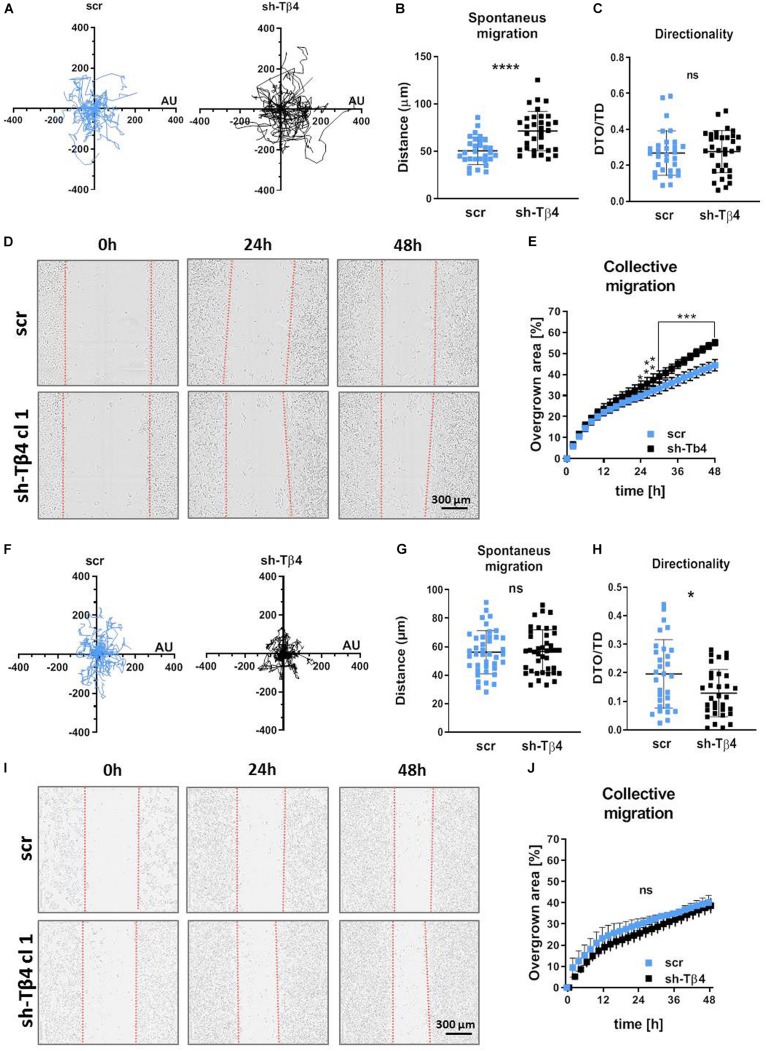
Decreasing of Tβ4 level affects A375 cells’ 2D motility. The cells were seeded either on non-coated surface **(A–E)** or on Matrigel^TM^ surface **(F–J)**. **(A–C,F–H)** Spontaneous 2D-migration of tested cells. The cells were recorded over 72 h (*n* = 30): **(A,F)** trajectories of single cells migration, **(B,G)** calculated migrated distances of the cells, **(C,H)** estimation of directionality of cells’ migration. Wound healing assay performed on tested cells (*n* = 3): **(D,I)** representative pictures of cells migrating collectively and **(E,J)** graphs representing scratch closure expressed in % of closed scratch. The significance level was set at ^∗^*P* < 0.05, ^∗∗^*P* < 0.01, ^∗∗∗^*P* < 0.001, and ^∗∗∗∗^*P* < 0.0001 (www.oncomine.org, February 2018, Thermo Fisher Scientific, Ann Arbor, MI, United States).

To determine if the differences in 2D migration potential of two tested melanoma cell lines are connected with Tβ4 level, we further tested A375-sh*TMSB4X* and A375-shscramble clones. 2D migration assay showed surprisingly that A375-sh*TMSB4X* clones covered significantly longer distances compared to control clone (Figures 5A,B). Directionality of protrusion on non-coated surface was unaffected by lowered Tβ4 level (Figure 5C). Interestingly, similarly to covered distances, collective migration was improved in A375-sh*TMSB4X* clones compared to control cells (Figures 5D,E and Supplementary Figure S12). Again we performed 2D migration analyses on Matrigel^TM^-coated surface. We found out that though the covered distances were indistinguishable for tested clones (Figures 5F,G), directionality of cells with lowered Tβ4 level was statistically significantly altered in comparison to control cells (Figure 5H). On the contrary, scratch was closed similarly fast by both tested types of clones (Figures 5I,J and Supplementary Figure S13).

### Tβ4 Influences Epithelial-Mesenchymal Transition in Melanoma Cells

**FIGURE 6:**
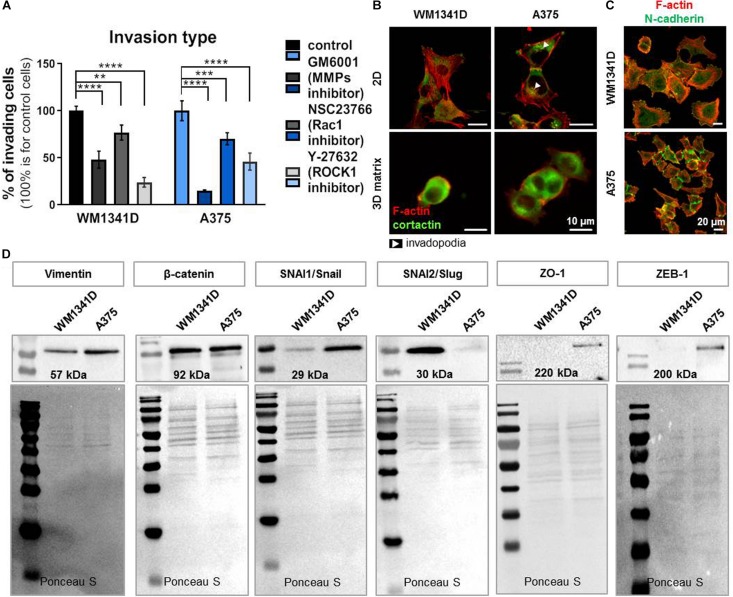
A375 cells are more progressed in epithelial-mesenchymal transition (EMT) than WM1341D cells. **(A)** Analysis of 3D motility type exhibited by studied cell lines by inhibitors application: 25 μM GM6001, 10 μM NSC23766, 10 μM Y-27632. The cells were seeded onto Matrigel^TM^ gel placed in the upper compartment of a Transwell^TM^ in medium with added inhibitors. 24 h later the assay was terminated (*n* = 3). **(B)** Immunostainings performed on cells growing under 2D or 3D (the cells were embedded in Matrigel^TM^ gel) conditions. F-actin and cortactin were visualized by application of fluorescently labeled phalloidin and antibodies recognizing cortactin. Arrowheads point at invadopodia. **(C)** Visualization of N-cadherin and F-actin in studied cells by immucytochemical staining. **(D)** Representative immunoblots of EMT markers levels in tested cell lines. 30 μg of protein was loaded on every lane. Membranes prior to incubation with antibodies were stained with Ponceau S to show equal protein loading on lanes. The significance level was set at ^∗^*P* < 0.05, ^∗∗^*P* < 0.01, ^∗∗∗^*P* < 0.001, and ^∗∗∗∗^*P* < 0.0001 (www.oncomine.org, February 2018, Thermo Fisher Scientific, Ann Arbor, MI, United States).

We analyzed the type of cell movement of both cell lines. WM1341D and A375 cells motility was impaired by addition of inhibitors such as GM6001 (matrix metalloproteinases inhibitor; Schultz et al., 1992), NSC23766 (inhibitor of Rac1; Gao et al., 2004) and Y-27632 a selective, ATP-competitive inhibitor of Rho-associated protein kinase (ROCK1) (Uehata et al., 1997). After treatment with GM6001 the number of invading cells was decreased by 50 and 85% for WM1341D and A375 cells, respectively. In the case of NSC23766 addition the number of cells with invasive potential was diminished by 25 and 30% for WM1341D and A375 cells, respectively. Addition of Y-27632 caused a drop in the number of invading cells by 75 and 50% for WM1341D and A375 cells, respectively (Figure 6A). Visualization of F-actin and cortactin (invadopodia marker; Artym et al., 2006) revealed invadopodia formation only in A375 cells (Figure 6B), while in 3D Matrigel^TM^ matrix we observed rounded morphology for WM1341D cells and more elongated shape for A375 cells (Figure 6B). These observations show that WM1341D cells move in a more ameboidal way, whereas A375 cells protrude in a more mesenchymal way. Generally, A375 cells were more prone to invade through the Matrigel^TM^ gel toward a chemotactic gradient than WM1341D cells (Figure 1D).

Next, we checked by immunostainings the presence of N-cadherin in studied cells, what corroborated our presumption that WM1341D and A375 underwent EMT (Figure 6C). N-cadherin is expressed in both cell lines in low and high density, however, we observed changes in localization. In a more confluent cell culture this protein is localized in adherens/tight junctions and in single cells the signal from staining is more dispersed (Supplementary Figure S14). The same situation we observed in β-catenin staining, however, in any case we did not notice translocation to nucleus. While we did not observe the presence of ZO-1 in adherens/tight junctions. Western blot analysis (WB) was performed to further estimate the levels of several EMT markers (proteins involved in cell’s epithelial or mesenchymal phenotype) such as vimentin, β-catenin, SNAI1/Snail, SNAI2/Slug, ZO-1, and Zeb-1 (Lamouille et al., 2014). Elevated levels of vimentin, SNAI1/Snail, ZO-1, and Zeb1 were observed in A375 cells when compared to WM1341D cells (Figure 6D). Interestingly, SNAI2/Slug was expressed at high level only in WM1341D cells (Figure 6D). On the other hand expression of β-catenin was similar in both cell lines (Figure 6D). Obtained results imply that tested cell lines are at different stages of EMT. In order to control the amount of loaded protein on lanes we performed total protein analysis (TPA) (Aldridge et al., 2008). The reasons for that are discussed elsewhere (Mazur et al., 2016a). In Table 1 we compared results obtained for WM1341D and A375 cell lines.

**TABLE 1:** Comparison of WM1341D and A375 melanoma cell lines studied features.

**Feature**	**WM1341D**	**A375**
*TMSB4X* expression level	Low	High
Invasion potential	Low	High
Type of cell movement	More ameboidal	More mesenchymal
2D Migration	Short distances	Long distances
Average number of focal adhesion (per cell)	24 h – 43 FAs 48 h – 80 FAs 72 h – 93 FAs	24 h – 20 FAs 48 h – 20 FAs 72 h – 20 FAs
Spreading area (average cell’s area)	49 AU	31 AU
Number of cells adhering to a substrate within a given time	High	Low
Advancement in EMT	Less progressed	More progressed

**FIGURE 7:**
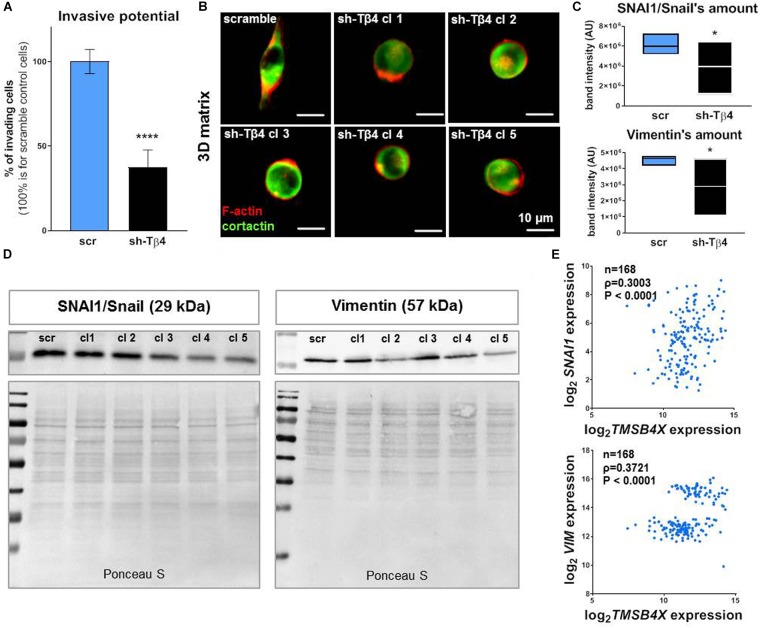
Silencing of *TMSB4X* expression has an impact on EMT in A375 cells. **(A)** Invasive potential of A375-sh*TMSB4X* clones compared to A375-*shscr* (*n* = 3). **(B)** Immunocytochemical analysis of A375-*shscr* and A375-sh*TMSB4X* clones embedded in Matrigel^TM^ gel. Fluorescently labeled phalloidin and antibodies were used to visualize F-actin and cortactin. **(C,D)** Western blot analysis of SNAI1/Snail and vimentin levels in A375-*shscr* and A375-sh*TMSB4X* clones: **(C)** densitometry (*n* = 3) and **(D)** representative immunoblots. 30 μg of protein was loaded on every lane. Membranes prior to incubation with antibodies were stained with Ponceau S to show equal protein loading on lanes. **(E)** Microarray analysis of *TMSB4X* and *SNAI1* or *VIM* expression’s correlation in patients samples (*n* = 168). The significance level was set at ^∗^*P* < 0.05, ^∗∗^*P* < 0.01, ^∗∗∗^*P* < 0.001, and ^∗∗∗∗^*P* < 0.0001 (www.oncomine.org, February 2018, Thermo Fisher Scientific, Ann Arbor, MI, United States).

Furthermore we decided to check what effect lowered Tβ4 levels had on melanoma cells in terms of invasion potential and EMT progression. We noted that decreased *TMSB4X* expression caused a reduction in invasion by more than 50% in A375-sh*TMSB4X* clones compared to control (Figure 7A). Simultaneously, we observed transition in cells’ shape in 3D matrix from a elongated one for A375-shscramble cells to a rounded one for A375-sh*TMSB4X* cells (Figure 7B). We noted also decreased expression level of SNAI1/Snail and vimentin at protein level (Figures 7C,D) and on mRNA level but only for SNAI1 (Supplementary Figure S15). Expression of other EMT markers remained unchanged (data not shown). Using microarrays data we show that both *SNAI1* (gene encoding SNAI/Snail) and *VIM* (gene encoding vimentin) expression levels exhibited positive correlation with *TMSB4X* expression in human melanoma patients (Figure 7E).

## Discussion

Melanoma cells are characterized by high heterogeneity due to its neuroectodermal origin and high plasticity. Cells during melanoma progression undergo phenotype switching, which manifests itself by the acquisition of mesenchymal and stem cells features (Kemper et al., 2014). We wanted to elucidate the role of Tβ4 in melanoma cell’s biology, since it was shown that in glioblastoma Tβ4 regulates invasion and stemness (Wirsching et al., 2014). In our study, we analyzed microarray data from melanoma patients and melanoma cell lines, which were characterized by a wide range of *TMSB4X* expression. For more detailed analysis we finally chose two cell lines originating from primary melanomas in vertical growth phase, i.e., WM1341D and A375 cells. To further analyse the functions of Tβ4 we obtained A375 stable clones with silenced *TMSB4X* expression.

Melanocytes and melanoma cells at early progression stages reside at the basement membrane, composition of which is resembled here in a Matrigel^TM^ form. Attachment to the basement membrane is loosened with disease progression. At the core of this process lay changes in the expression level of proteins involved in cell’s adhesion (Christofori, 2003; Saladi et al., 2010). We show here for the first time that Tβ4 is a component of FAs and is able to create complexes with FAs’ structural and signaling proteins, i.e., vinculin, ILK, FAK, pinch 2, α parvin and integrin αVβ3. It has been proven earlier that Tβ4 directly interacts with ILK and forms a functional complex with ILK-PINCH-Parvin in the cardiac cells (Bock-Marquette et al., 2004). It is for future studies to check if Tβ4 can directly interact with vinculin, FAK and integrin αVβ3. Sosne et al. (2002) presented data showing Tβ4 impact on FAs morphology in human conjunctival epithelial cells. Here, in comparison to WM1341D cells, the A375 cell line, characterized with high *TMSB4X* expression level, exhibited differences in FAs’ number, morphology and size. The lower number of big-sized FAs in A375 resulted in poorer cells’ spreading and adhesion ability to non-coated and Matrigel^TM^-coated surfaces in comparison to WM1341D cells exhibiting many small-sized FAs. We show for the first time that down-regulation of Tβ4 expression in melanoma cells resulted in an increased number of small-size FAs, which were smaller when compared to control cells. As a consequence A375-sh*TMSB4X* cells flattened and adhered better to Matrigel^TM^-coated surface. What is intriguing in this story is the fact that HA-Tβ4:vinculin, HA-Tβ4:ILK and HA-Tβ4:FAK complexes were detected in the whole body of analyzed cells differently to the localization of HA-Tβ4:pinch 2, HA-Tβ4:α parvin and HA-Tβ4:integrin αVβ3 complexes. It cannot be excluded that high Tβ4 abundance in cytoplasm prevents vinculin, ILK and FAK from targeting plasma membrane or participation in FAs assembly. In cell’s movement the important role in force generation plays adhesion to ECM proteins. We show that A375 cells characterized by high *TMSB4X* expression were more advanced in melanoma progression than WM1341D cells. It was supported by greater motility of A375 cells in 2D and 3D Matrigel^TM^ matrix. Although the differences between WM1341D and A375 cells moving on Matrigel^TM^-coated surface were more modest when compared to motility of the cells on non-coated surface. WM1341D line is characterized by a higher number of FAs and it seems that for these cells interaction with surface is more important. Their migration speed as well adhesion abilities are increased when surface is coated with Matrigel^TM^. It highlights again the fact that choosing proper conditions for performing experiments is very important, and in some cases plastic, as a non-physiological material, may influence results. We observed also other mesenchymal features such as invadopodia formation and elongated morphology in 3D Matrigel^TM^ matrix.

Surprisingly, decreased Tβ4 level resulted in increased 2D migration on non-coated surface. Nevertheless, motility on Matrigel^TM^-coated surface of A375 cells with lowered Tβ4 level was impaired. Distances covered by control and A375-sh*TMSB4X* clones were similar but directionality of A375-sh*TMSB4X* cells was severely affected. The directional cell movement is based on two main mechanisms, i.e., chemotaxis and haptotaxis. The former depends on the chemical gradient and is quite well known. The latter, responsible for directionality of movement in response to adhesive substrates such as ECM is far less understood. The comparison of migratory behavior of mesenchymal stem cells and fibroblasts revealed that reducing cell’s interactions with the surface lead to haptotactic directional cell movement. Moreover, too strong or too weak binding to surface prevented this type of movement. Additionally, it was shown that cells ability to spread on a surface may play a role in limiting haptotaxis (Wen et al., 2015). Here we observed a similar phenomenon. In A375-sh*TMSB4X* cells we observed increased spreading ability, followed by reduced directionality of movement. In breast cancer cells, this type of migration is based on interaction between α5β3 integrin and actin regulator protein – Mena. Expression of this protein in cancer cells is responsible for directional cell movement via its interaction with α5 integrin subunit and F-actin (Oudin et al., 2016). While in melanoma cells integrin αVβ3 plays a role in haptotactic motility (Aznavoorian et al., 1996). What’s intriguing we show here that Tβ4 probably forms complexes with αVβ3 integrin. However, further studies are required to understand the role of this polypeptide in directionality of cell migration. We did not observe statistically significant differences between WM1341D and A375 cells in terms of directionality. Cells with lowered Tβ4 level shared some aspects of phenotype with WM1341D cells but not all. For instance they differed in manifestation of EMT markers. Possibly they may also differ in activation of signaling pathways. We have not looked at signaling in tested cells as it is beyond the scope of this study. But it could be interesting to check, in the future, the involvement of two axes playing a role in cells motility directionality, i.e., Rac/Par3, Par6, and Cdc42, LPA receptor/Rab5 or N-cadherin recycling (Scarpa and Mayor, 2016). These observations imply as well that surface coating (thin layer of polymerized Matrigel^TM^ spread over a coverslip or well bottom) is a key factor in motility assays and finally Tβ4 influences cells protrusion on resembling basement membrane composition. That is why it is not surprising that invasion of cells with lowered Tβ4 level was impaired. Diminished 3D migration is hence plausible, since it was previously shown that increased Tβ4 level in endothelial cells induced expression of matrix metalloproteinases (Cierniewski et al., 2007), which are crucial for mesenchymal type of movement in 3D matrix.

A375 cells were more advanced in EMT than WM1341D cells, which was corroborated by high vimentin, Zeb1, and SNAI1/Snail (EMT markers) expression. At the same time we observed a higher level of ZO-1 in A375 cells than in WM1341D cells. Recent studies suggest that EMT in cancer cells is a dynamic and reversible process manifested by the spectrum of intermediate states between epithelial and mesenchymal phenotypes. It was shown that tumor cells with “hybrid” epithelial/mesenchymal phenotype are more invasive than fully mesenchymal (Pastushenko et al., 2018). Furthermore, ZO-1 (a marker of tight junctions in epithelial cells) and ADAM12 (a marker of mesenchymal cell differentiation) are co-expressed and interact with each other in invasive breast cancer cells. ZO-1 was localized in invadopodia-like structures and decreased expression of this protein suppressed matrix degradation and invasion of cells (Dekky et al., 2018). Here, visualization of ZO-1 in tested melanoma cell lines revealed a similar phenomenon. In A375 cells characterized by high invasion potential we did not observe ZO-1 in adherens/tight junctions. However, ZO-1 colocalized with F-actin in cellular protrusions involved in cell movement such as invadopodia and lamellipodia. In our studies we focused on melanoma cells which are tumor-transformed melanocytes, cells of neuroectodermal origin. In these cells the EMT may proceed differently compared to epithelial cells. To our current knowledge, there has not been performed any research about “hybrid” epithelial/mesenchymal phenotype in melanoma. However, there are reports showing highly heterogeneous expression of proteins associated with EMT in series of early passage human melanoma cell lines and melanocytes (Kim et al., 2013) as well as in histological studies on human melanoma tissue (Shirley et al., 2012). Intermediate EMT states in melanoma cells may play an important role in metastasis and should be more deeply investigated. Though SNAI1/Snail and SNAI2/Slug are considered to play the same biological role in EMT triggering in epithelial tumors (Lamouille et al., 2014), it has been shown for melanoma cells that SNAI2/Slug is responsible for melanocytic differentiation and thus suppresses EMT (Caramel et al., 2013). That is why it is not surprising that SNAI2/Slug was barely detectable in A375 cells. Lowering of Tb4 level resulted in partially reversed EMT in A375-sh*TMSB4X* clones, since we observed decreased level of only two EMT markers tested by us.

It was shown for hepatoblastoma and oral squamous cell carcinoma that Tβ4 induced EMT transition (Fu et al., 2015; Hong et al., 2016). Here, we demonstrate that also in melanoma cells there is a connection between *TMSB4X* expression and EMT progression. In colorectal carcinoma it was demonstrated that Tβ4 triggers EMT transition by ILK upregulation (Huang et al., 2007). Recent studies indicate that SNAI1/Snail regulates the expression of αV integrin and reduces the expression of ECM proteins in normal and tumor cells (Haraguchi et al., 2008), whereas FAK was identified in embryonic cells undergoing EMT as a regulator of SNAI1/Snail (Li X. Y. et al., 2011). These findings indicate the dependence of EMT on adhesion abilities and our results suggest a linking role of Tβ4 in it.

**FIGURE 8:**
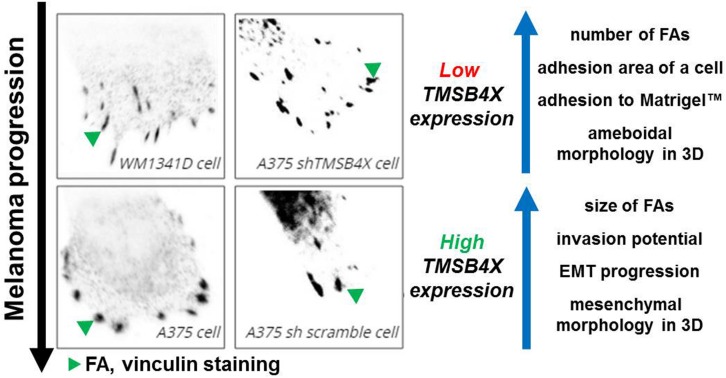
Influence of Tβ4 on focal adhesions’ formation and its impact on melanoma progression. Increased *TMSB4X* expression is positively correlated with FAs size, cells’ invasion abilities and expression of EMT markers. On the other hand we observed negative correlation between *TMSB4X* expression and number of FAs, cell’s adhesion area and ability of studied cells to adhere to ECM (Matrigel^TM^). Motility of cells with low expression of *TMSB4X* was more depended on Rac1 action than Rho/ROCK1 activity.

Ray et al. (2017) demonstrated that mesenchymal cells are characterized by large FAs and directional movement, while ameboid cells by small FAs and random migration. In our study we observed that cells with silenced *TMSB4X* expression were characterized with withdrawal of some properties of mesenchymal cells and dramatic alterations in FAs formation and adhesion abilities. Finally, analysis of patients’ microarray data supported some of our observations, there is a positive correlation between expression of *VIM* and *TMSB4X* and *SNAI1* and *TMSB4X*. We propose that Tβ4, by direct regulation of FAs formation, alters adhesion abilities of melanoma cells (Figure 8). This could lead to acquisition of mesenchymal features resulting in increased metastatic potential. Further studies should focus on unveiling Tβ4’s role in FAs dynamics, elucidation of character of Tβ4 interactions with FAs’ compounds and Tβ4 impact on the parameters studied here in aspect of different ECM proteins. It appears that Tβ4 is an important player in melanoma progression and could be potentially a target for anti-melanoma treatment.

## Materials and Methods

### Cell Lines and Culture Conditions

All tested cell lines were of human origin and were authenticated by ATCC^®^ in 2017 and 2018. A375 (CRL-1619^TM^) and Hs294T (HTB-140^TM^) cell lines were obtained from the ATCC^®^. WM1341D and WM9 cell lines were bought from Rockland Immunochemicals. The cell culture conditions are described elsewhere (Makowiecka et al., 2016).

### Invasion Assay

The assay is described elsewhere (Makowiecka et al., 2016). In some experiments 25 μM of a non-selective inhibitor of metalloproteases (GM6001, Santa Cruz Biotechnology Inc.), 10 μM Rac1 inhibitor (NSC 23766, Santa Cruz Biotechnology Inc.) or 10 μM ROCK1 inhibitor (Y-27632, Santa Cruz Biotechnology Inc.) were added to the medium, in which cells were seeded.

### Cell Migration Assay

2D spontaneous migration was monitored using IncuCyte^®^ ZOOM System (Essen BioScience). 1000 cells per well were seeded into 96-well IncuCyte^®^ ImageLock plates (Essen BioScience) and image sets have been collected every 2 h for 72 h. The single cell distance and trajectory have been analyzed using Manual Tracking plug-in for ImageJ (F. Cordelieres, Institute Curie, Paris, France) for 10 cells from three separate experiments. Directionality was calculated as it is described elsewhere (Masuzzo et al., 2017).

Wound healing assay was performed as described. Cells were seeded onto Essen Bioscience 96-well ImageLock plates and cultured until confluence was reached. With the use of IncuCyte^®^ WoundMaker (96-pin woundmaking tool) unified scratches were made. Collective cell migration was performed with the use of IncuCyte^®^ Live Cell Analysis Imaging System (with data collection every 2 h over 48 h) and analyzed with IncuCyte^®^ software. Results were presented as a percent of scratch overgrown area in time. To establish if Wound Maker is able to remove Matrigel^TM^ from coated surface, a confluent monolayer of cells growing on Matrigel^TM^ coated wells was scratched. Cells and surfaces of wells were immunostained using anti-pan-laminin antibodies and fluorescently labeled phalloidin in order to visualize laminin and F-actin.

### Immunocytochemistry and Confocal Microscopy

For details see Makowiecka et al. (2016). Used antibodies and their dilutions are listed in Supplementary Table S1, Alexa Fluor^®^ 568-labeled phalloidin and Hoechst 33342 were purchased from Invitrogen. Photos and Z-stacks were taken using the Olympus FluoView 500 and Zeiss LSM510 confocal laser scanning microscopes. The number of FAs was semi-automatically calculated using ImageJ software, according to a procedure described elsewhere (Horzum et al., 2014). The cell area was analyzed using ImageJ. A super-resolution confocal microscope (Zeiss LSM880 with Airyscan module) was applied to collect some of the data. Immunostaining in 3D matrix was performed on cells seeded into 96-well plates in 2 mg/ml Matrigel^TM^ solution in medium without FBS.

### Western Blot Analysis

The procedure describing the preparation of cell lysates and WB is described elsewhere (Makowiecka et al., 2016). 30 μg of protein was loaded on every lane. Prior to the blocking step the membranes were stained in 0.2% Ponceau S solution for 10 min for TPA. Used antibodies and their dilutions are listed in Supplementary Table S1. Densitometric analysis of the membranes was performed with the Image Lab 4.0 software (Bio-Rad). From three separate WBs performed for each protein the volume intensity of band (the intensity of luminescence signal in the whole volume of the detected protein band) was measured.

### Quantitative Polymerase Chain Reaction (qPCR)

RNA from cells was isolated using GenElute^TM^ Mammalian Total RNA Miniprep Kit (Sigma-Aldrich) and the DNA was digested with DNase I (Sigma-Aldrich) according to manufacturer’s protocol. cDNA was obtained by reversed transcription of 0.5 μg total RNA using High Capacity cDNA Reverse Transcription Kit (Applied Biosystems). For qPCR reaction PowerUp^TM^ SYBR^TM^ Green Master Mix (Thermo Fisher Scientific) was used according to manufacturer’s protocol. Applied Biosystems StepOne^TM^ was used for qPCR performance. For quantification, the samples were normalized against the expression of a housekeeping gene – *HPRT1* (hypoxanthine phosphoribosyltransferase 1). Primers are listed in Supplementary Table S2.

### DNA Constructs

PCR products were cloned into a vector using NEBuilder HiFi DNA Assembly Master Mix (New England Biolabs). Primers and vectors are listed in Supplementary Table S3. Plasmid pLVX-IRES-tdTomato-FlagAkt1 was a gift from Eva Gonzalez (Addgene plasmid # 64831) (Kajno et al., 2015) and plasmid p3xHA-C1 (Müller et al., 2012). The accuracy of DNA constructs was verified by sequencing. Plasmids for *TMSB4X* gene silencing were obtained from Sigma-Aldrich – five MISSION pLKO.1-puro *TMSB4X* shRNA vectors and a control MISSION pLKO.1 puro Non-Target shRNA. Sequences are listed in Supplementary Table S4.

### Cell Transfection

The cells were transfected with expression vectors using 2 mg/ml polyethyleneimine (PEI) solution (Sigma-Aldrich). In order to introduce shRNA vectors into cells Neon Transfection System (Thermo Fisher Scientific) was used. Applied pulse parameters were: 1100 V, 40 ms. Stable cell lines were obtained by 1 μg/ml puromycin selection.

### Proximity Ligation Assay

The assay was performed according to manufacturer’s protocol (Sigma-Aldrich). For details see Mazur et al. (2016b). Photos were taken with the use of Zeiss LSM510 confocal microscope. Number of positive signals were quantitatively analyzed in 20 cells per group using ImageJ software equipped with appropriate plugin (Horzum et al., 2014). The protocol was modified as follows: Log3D plugins parameters sigma *X* = 3 and sigma *Y* = 3 were used and for ANALYZE PARTICLES command following parameters were used: size = 0–infinity and circularity = 0–1.0.

### Adhesion Assay

The assay was performed as described elsewhere (Radwanska et al., 2012). 35000 cells were seeded into 96-well plates with 2 μg/cm^2^ Matrigel^TM^ or without coating surface in serum-free medium containing 0.5% BSA, 2 mM MgCl_2_ and 2 mM CaCl_2_. After incubation (1 h at 37°C) unbound cells were removed by five D-PBS washes and the adhered cells were analyzed with a MTT assay (Sigma-Aldrich) according to manufacturer’s protocol.

### Data Preprocessing of Microarray Gene Expression

Gene expression data of melanoma patients on three Affymetrix human genome microarray platforms (U133A, U133A2, U133APlus2) were obtained from Gene Omnibus (GEO). The datasets included: GSE3189 (*n* = 70), GSE4587 (*n* = 15), and GSE8401 (*n* = 83). Each data set was background corrected and normalized using Robust Multichip Average algorithm (RMA) and subsequently compiled and adjusted for batch effect using ComBat (Johnson et al., 2007). Probes for *TMSB4X*, *SNAI1*, and *VIM* were selected with Jetset algorithm (Li Q. et al., 2011). Preprocessed data sets consisted totally of nine normal skin samples, 22 nevi-like skin samples, 82 from primary, and 55 from metastatic melanoma biopsies. Non-parametric test (Spearman’s rank correlation) was used to estimate the correlation and significance of *TMSB4X* with other genes co-expression.

### Statistical Analysis

All data are given as means ± standard deviations (SD) are representative of at least three independent experiments. Their significance was determined using either the two-tailed, unpaired Student’s *t*-test or ANOVA (one-way or two-way) with *post hoc* Tukey HSD were applicable, which were performed in GraphPad Prism 7. The significance level was set at ^∗^*P* < 0.05, ^∗∗^*P* < 0.01, ^∗∗∗^*P* < 0.001, or ^∗∗∗∗^*P* < 0.0001. Graphs were plotted in GraphPad Prism 7.

## Conclusion

Thymosin β4 is a predominantly expressed thymosin β in human melanoma cells and is a constituent of FA. This polypeptide regulates formation of FAs in melanoma cells by influencing their morphology and number. Moreover, it has significant impact on adhesion and protrusive abilities under 2D and 3D conditions of melanoma cells. Tβ4 level decides about progression of EMT of melanoma cells.

## Data Availability Statement

The raw data supporting the conclusions of this article will be made available by the authors, without undue reservation, to any qualified researcher.

## Author Contributions

AM partially designed the study, performed the experiments, contributed to the preparation of the figures, and took part in writing the manuscript. NM, EMa, and EMr performed some experiments and contributed to the preparation of the figures. AJM designed the study, gained funding for the study, performed the experiments, contributed to the preparation of the figures, and took part in writing the manuscript. DN discussed the manuscript. All authors reviewed the results and approved the final version of the manuscript.

## Conflict of Interest

The authors declare that the research was conducted in the absence of any commercial or financial relationships that could be construed as a potential conflict of interest.

## Footnotes

1 www.oncomine.org
