# Supplementary material for: Thymosin β4 Regulates Focal Adhesion Formation in Human Melanoma Cells and Affects Their Migration and Invasion
Source: Front Cell Dev Biol. 2019 Dec 23;7:304. doi: 10.3389/fcell.2019.00304 (PMC6935720; doi:10.3389/fcell.2019.00304)
Supplement: Supplementary file 1 [file Data_Sheet_1.pdf]

Supplementary Material

1 Supplementary Figures and Tables

1.1 Supplementary Figures

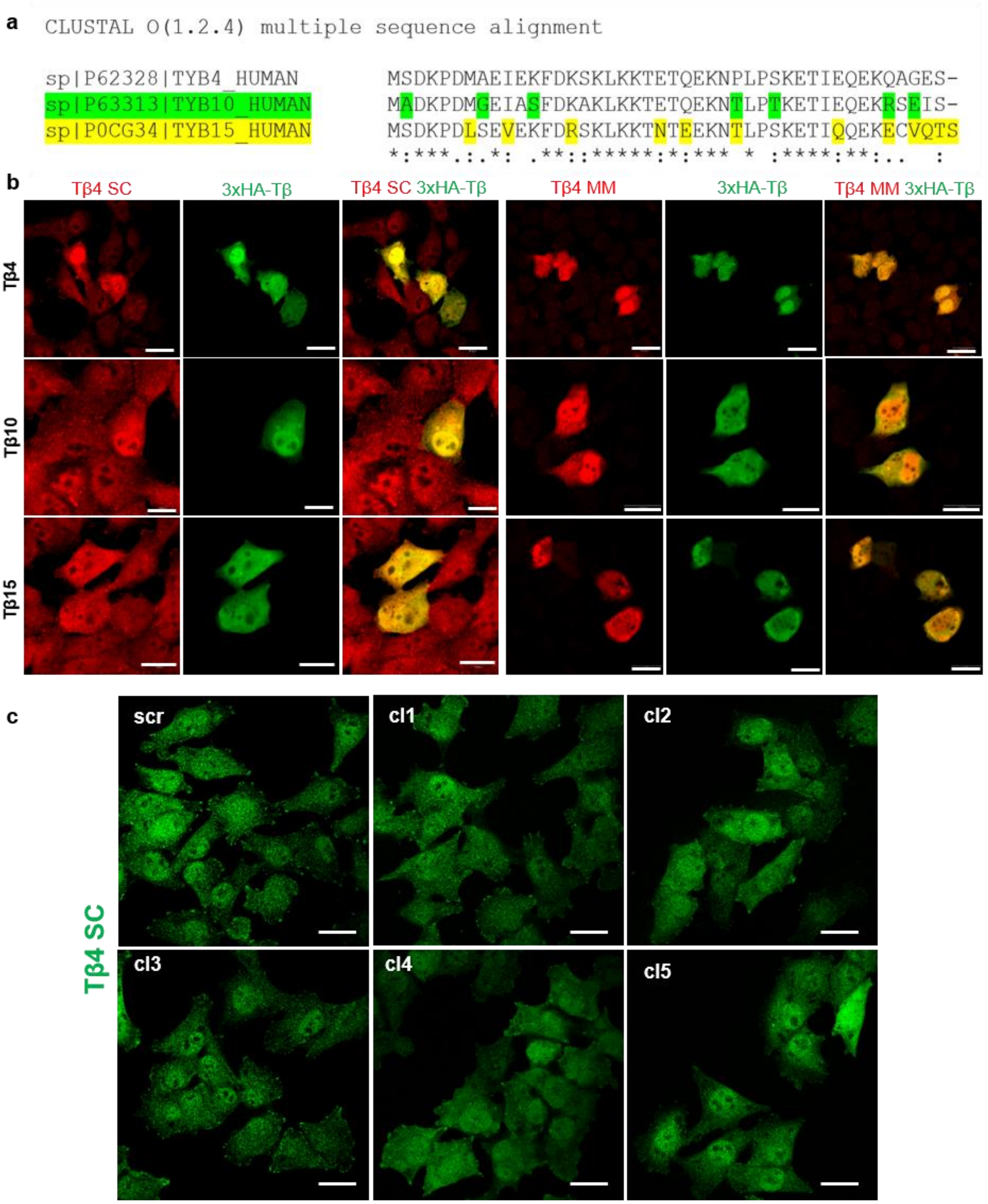

Supplementary Figure 1. (a) Multiple sequence alignment in Clustal Omega [53] of β thymosins (Tb4, Tb10 and Tb15) amino acid sequences with the help of BLAST online tool (Sequences were taken from Uniprot database). **(b)** Analysis of antibodies' specificity against Tβ4 (Table S1. List of

antibodies). The A375 cells were transfected with plasmids encoding Tβ4, Tβ10 or Tβ15 tagged with 3xHA and upon fixation immunostained with anti-HA and two anti-Tβ4 antibodies. Scale bar = 20 µm. SC: antibodies from Santa Cruz Biotechnology Inc. MM: antibodies from Merck-Millipore. (c) Immunocytochemical analysis of A375-sh*TMSB4X* and A375-shscramble clones with anti-Tβ4 antibodies (SC). Scale bar = 20.

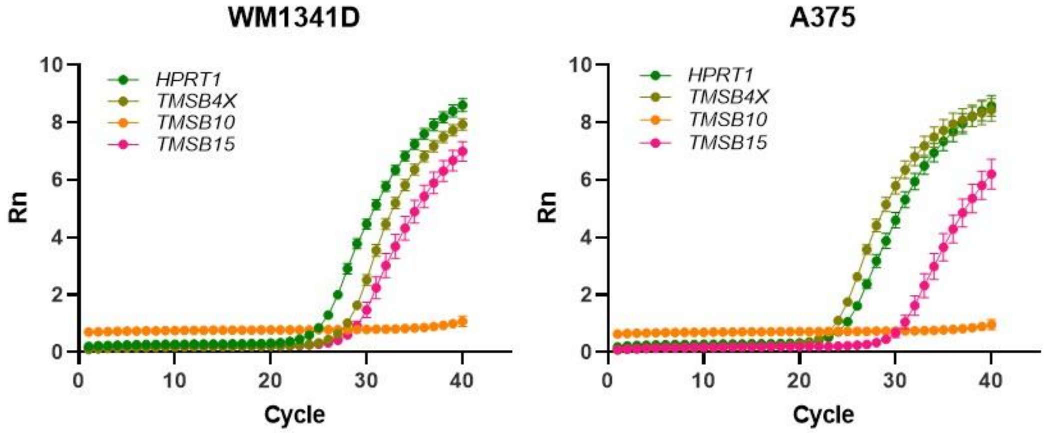

Supplementary Figure 2. Quantitative RT-PCR amplification curves for *HPRT1*, *TMSB4X*, *TMSB10* and *TMSB15* obtained from cDNA of WM1341D (a) and A375 (b) cells.

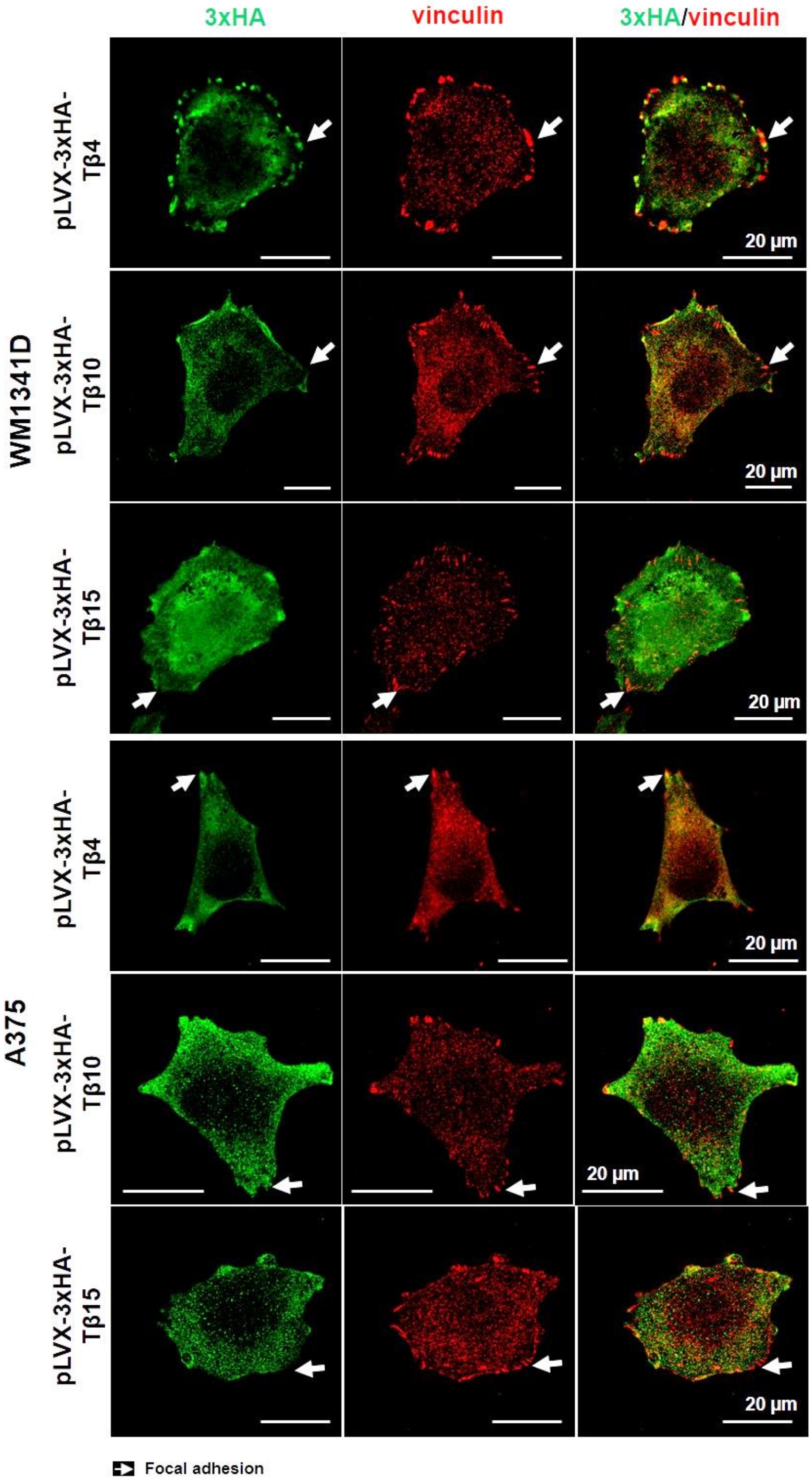

Supplementary Figure 3. Visualization of vinculin and 3xHA-Tβ4, 3xHA-Tβ10 or 3xHA-Tβ15 respectively in WM1341D and A375 cells transfected with pLVX-IRES-puro-3xHA-Tβ4, pLVX-IRES-puro-3xHA-Tβ10 or pLVX-IRES-puro-3xHA-Tβ15. Arrows point at focal adhesions. Scale bar = 20 μm.

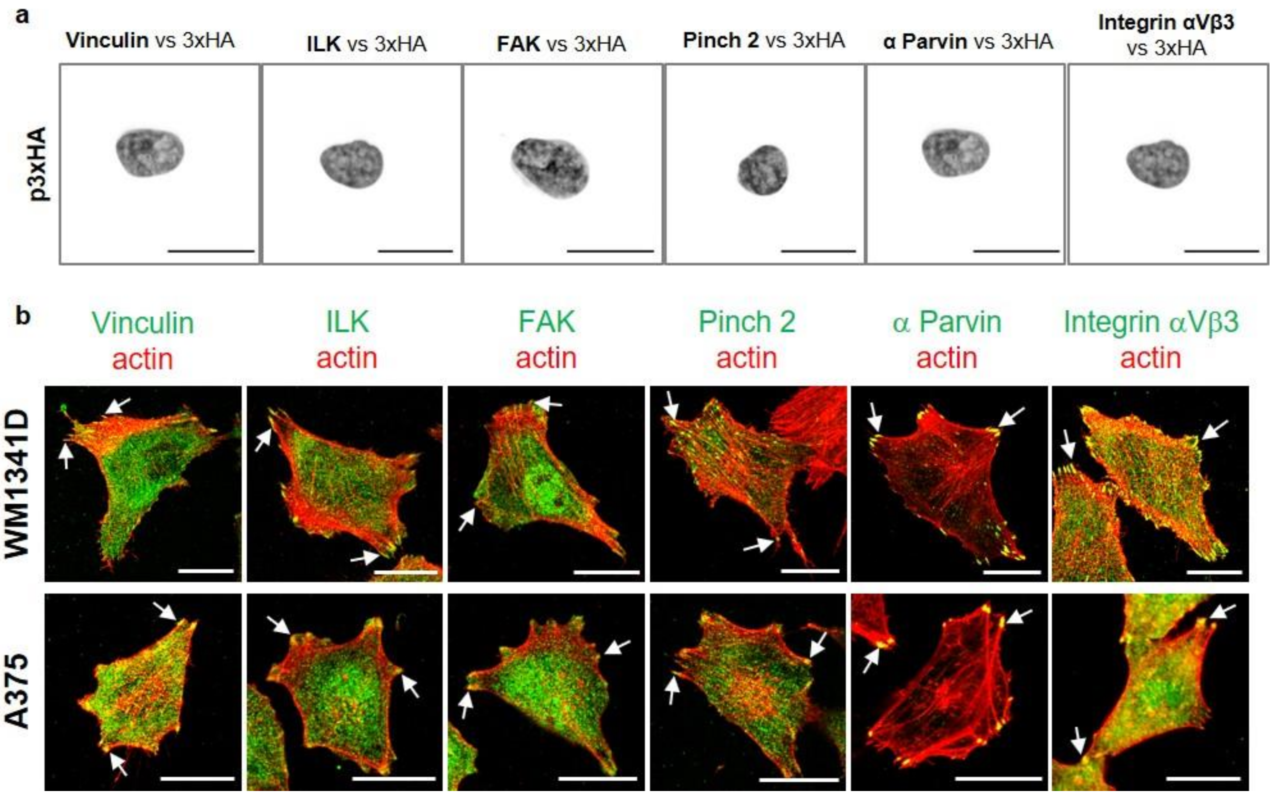

Supplementary Figure 4. (**a**) Negative controls for PLA assay. (**b**) Visualization of vinculin, ILK, FAK, a parvin, Pinch 2 and integrin aVb3 in WM1341D and A375 cells. The cells were additionally stained with antibodies recognizing all isoactins (clones AC40 or C11) or fluorescently labeled phalloidin. Arrows point at focal adhesions. Scale bar = 20 µm.

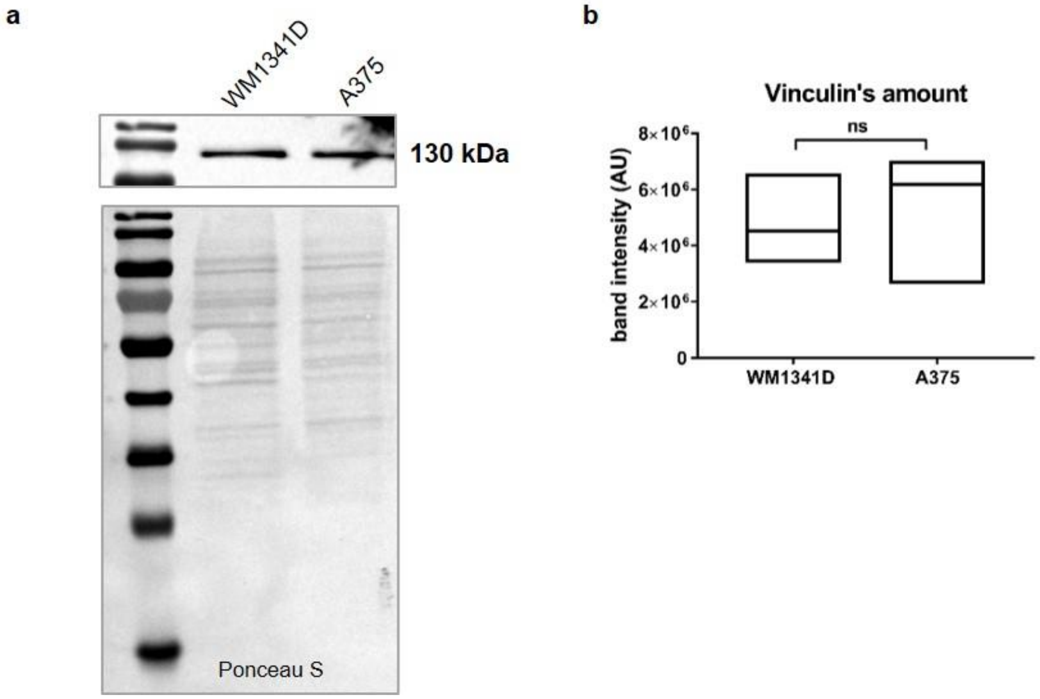

Supplementary Figure 5. (a-b) Analysis of vinculin level in WM1341D and A375 cell lysates: **(a)** a representative immunoblot and Ponceau S staining of the whole membrane, **(b)** densitometrical analysis (n=6).

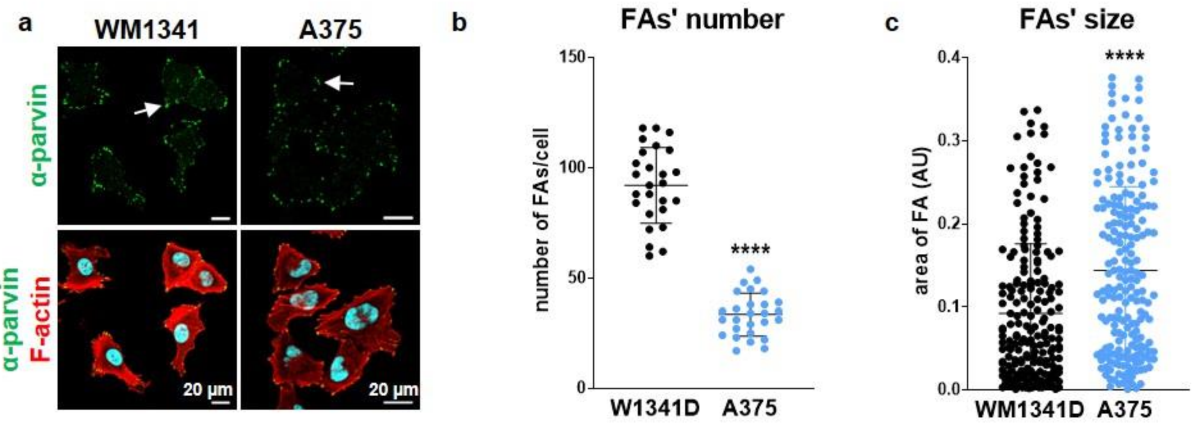

Supplementary Figure 6. Analysis of FAs' formation in tested cell lines: Visualization of a-parvin and F-actin in WM1341D and A375 cells fixed 24 h after seeding the cells **(a)**. Estimation of FAs' number **(b)** and size of FA's **(c)**. **** denotes significant difference between marked groups. Arrows point at focal adhesions. Scale bar = 20 µm.

a

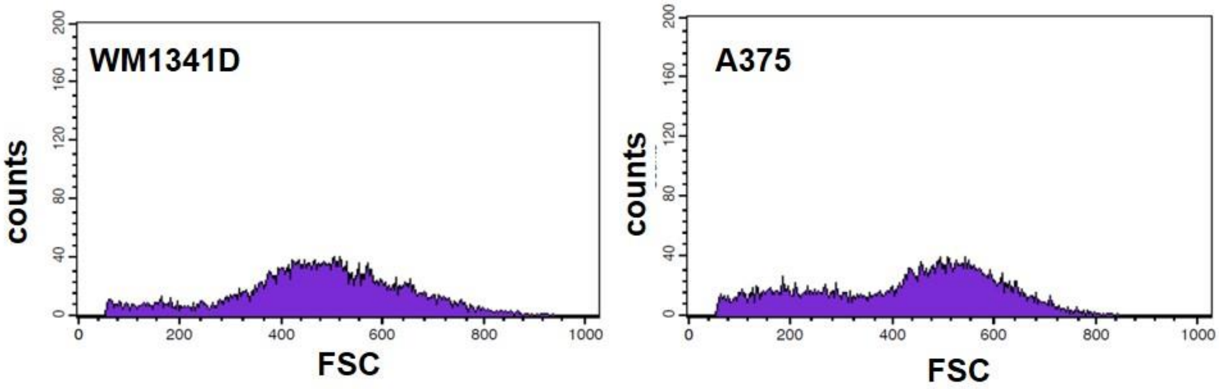

b

cell line	events	mean	geo mean	median
WM1341D	10000	471	436	474
A375	10000	423	375	463

Supplementary Figure 7. Analysis of cells' size (Forward scatter - FSC). Cells were analyzed with a BD FACS Calibur Flow Cytometry System using BD CellQuest Pro Software; cells were washed three times with cation-free PBS supplemented with 2 mM EDTA and then transferred to a FACS tube (n=3). Cells were gated on single living cells. The number of analysed cells in every experiment was 10000. **a)** Histogram showing distribution of cells' size in WM1341D and A375 cell lines. **b)** Histogram statistic.

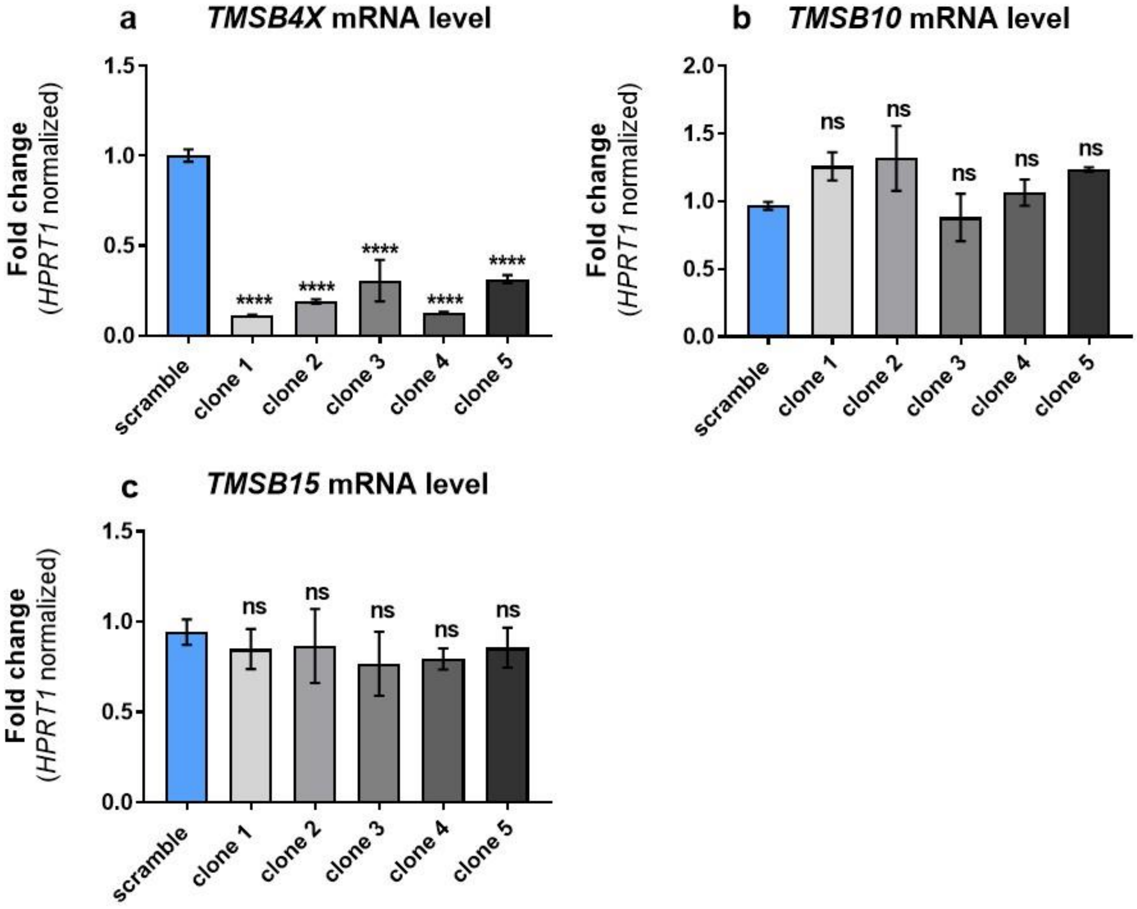

Supplementary Figure 8. qRT-PCR analysis of *TMSB4X* **(a)** *TMSB10* **(b)** and *TMSB15* **(c)** expression level in A375-shscr and A375-*shTMSB4X* clones.

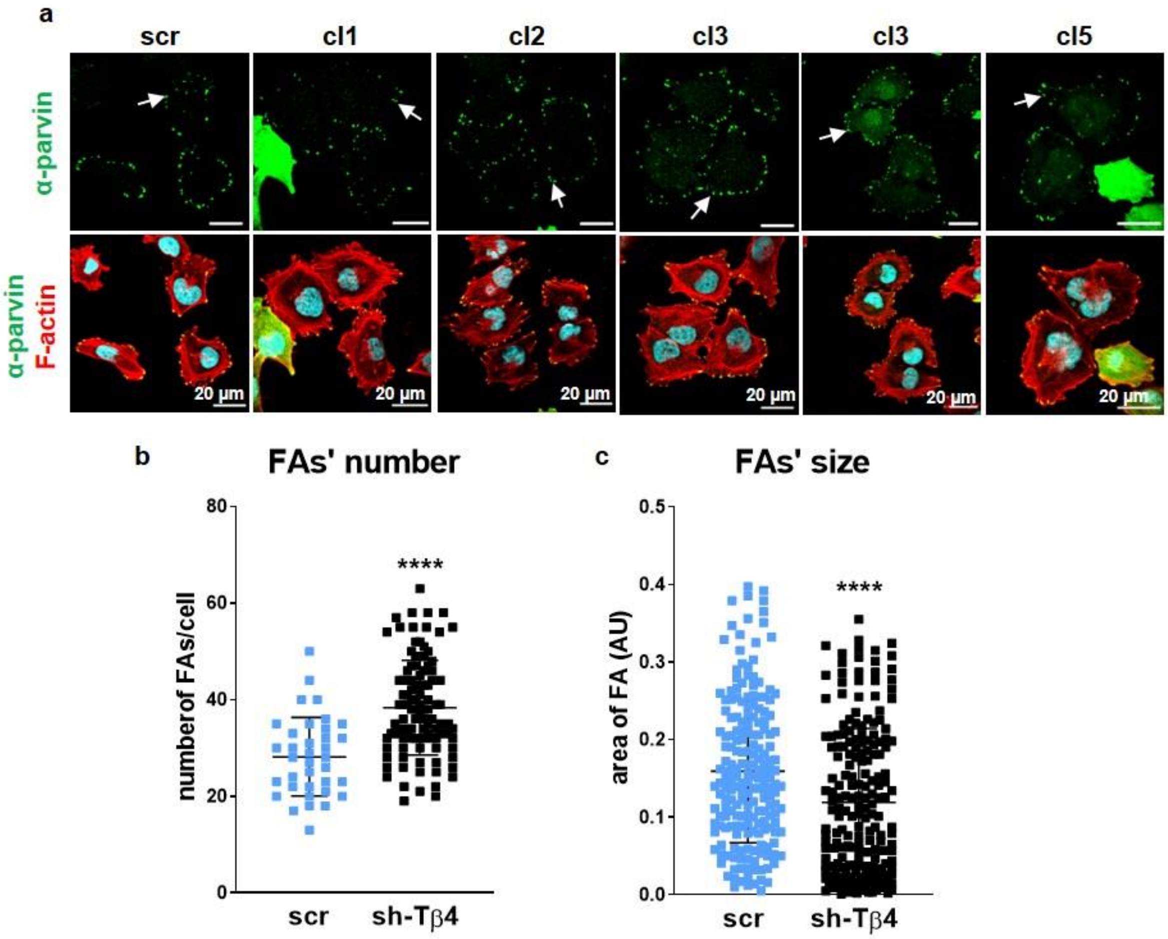

Supplementary Figure 9. Analysis of FAs' formation in tested cell lines: Visualization of a-parvin and F-actin in A375 cells with lowered *TMSB4X* expression. **(a)** Estimation of FAs' number **(b)** and size of FA's **(c).** ****denotes significant difference between marked groups. Arrows point at focal adhesions. Scale bar = 20 µm.

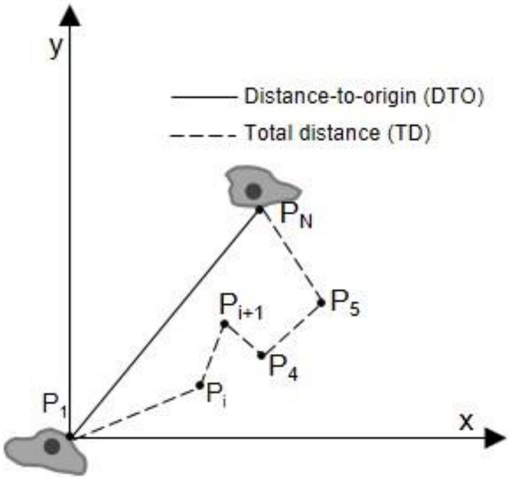

Supplementary Figure 10. Single cell trajectory in 2D space with quantitative parameters defining directionality. Directionality ratio from the start point (P1) to the end-point (PN) of the cell trajectory is calculated by dividing the displacement (distance to origin, DTO) and total length of trajectory (total distance, TD).

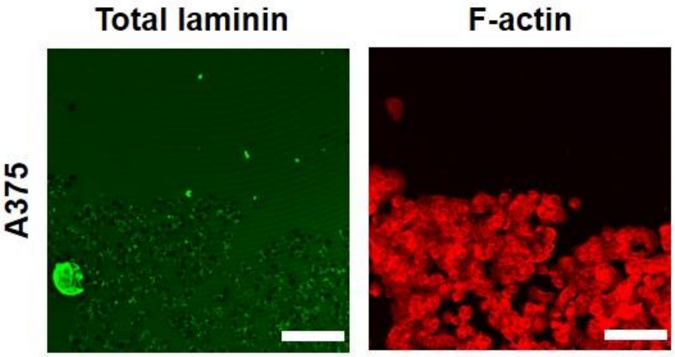

Supplementary Figure 11. Analysis of Matrigel® coated wells after scratching. Cells and surfaces of wells were immunostained using anti-laminin antibodies and counterstained with fluorescently-labeled phalloidin. Wound Maker did not remove Matrigel® from surfaces of wells. Scale bar = 200 μm.

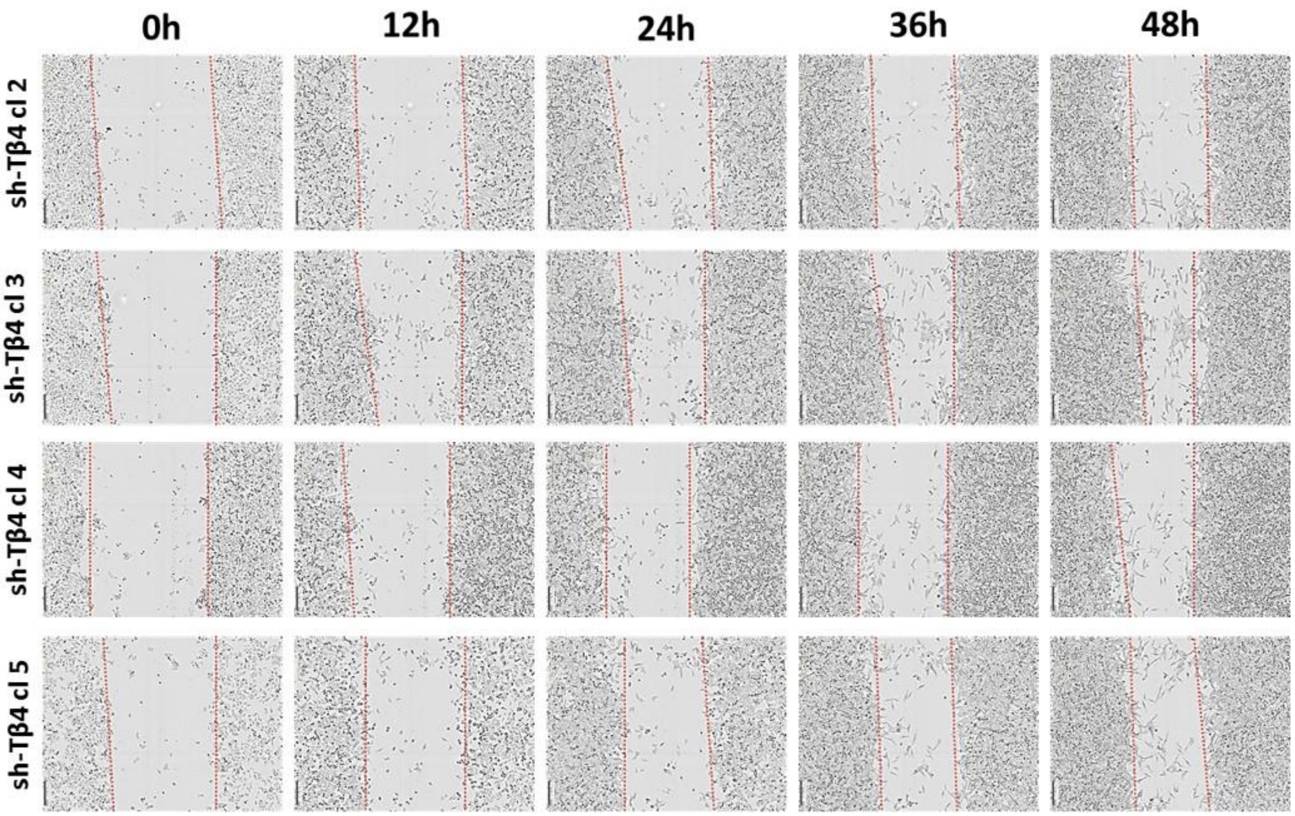

Supplementary Figure 12. Evaluation of Tβ4 role in A375 clones with decreased Tβ4 level. Representative pictures of cells migrating collectively on plastic recorded over 72h for A375-*shTMSB4X* clones (n=3).

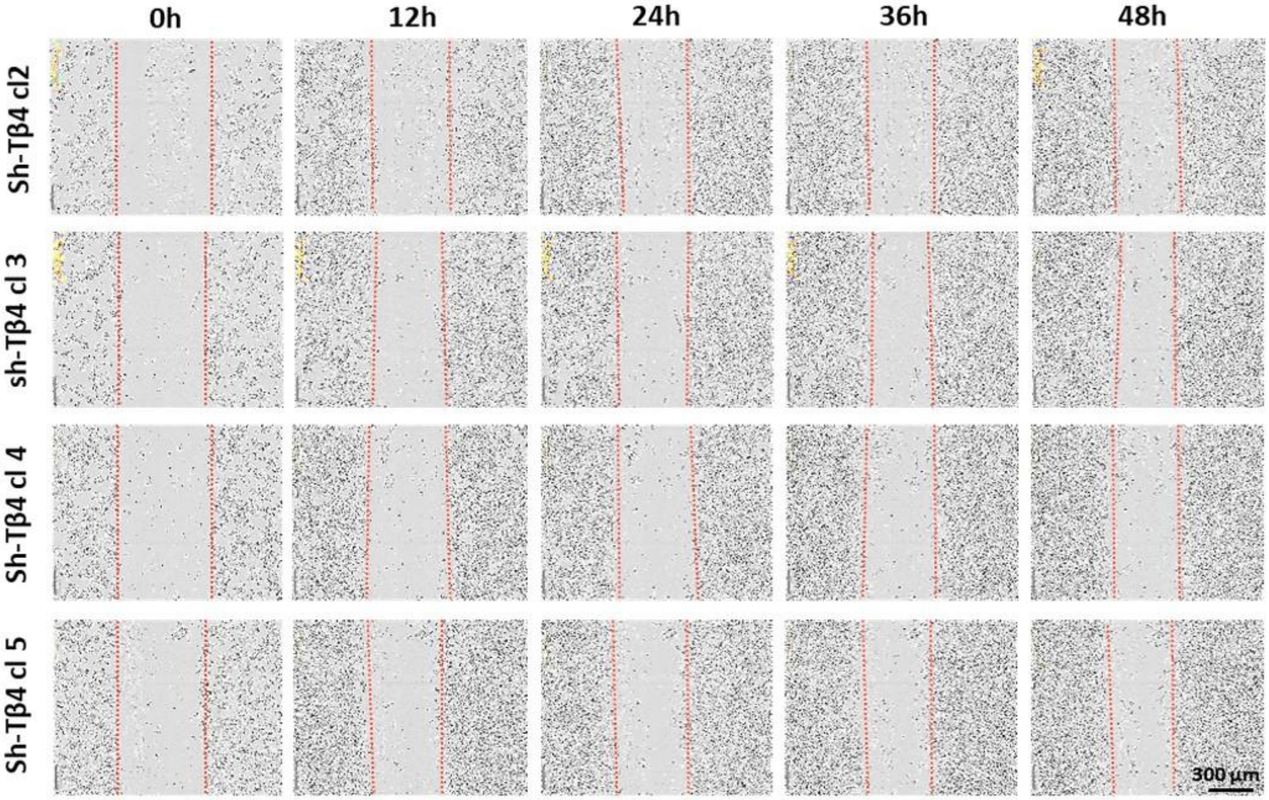

Supplementary Figure 13. Evaluation of Tβ4 role in A375 clones with decreased Tβ4 level. Representative pictures of cells migrating collectively on Matrigel™ recorded over 72h for A375-*shTMSB4X* clones (n=3).

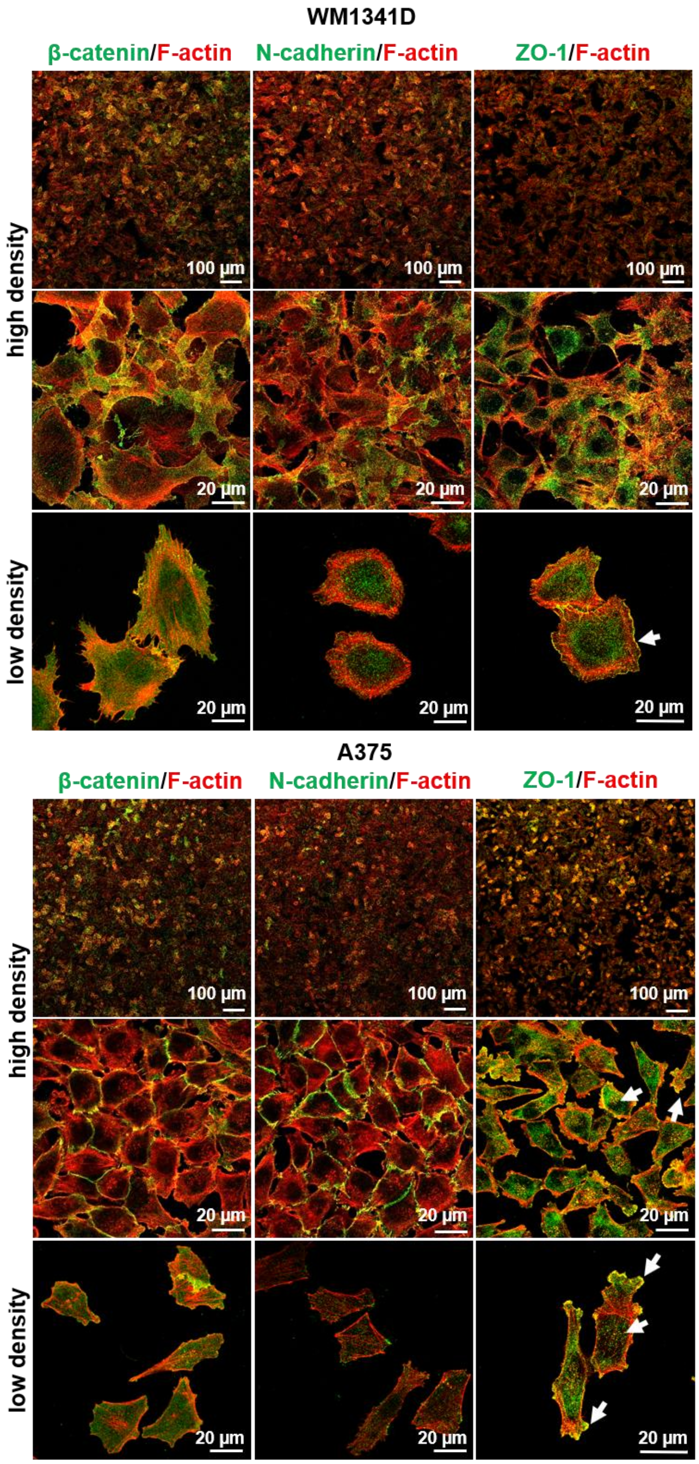

Supplementary Figure 14. Visualization of β-catenin, N-cadherin and ZO-1 in WM1341D and A375 cells seeded at different densities. The cells were additionally stained with fluorescently labeled phalloidin. Arrows point at cellular protrusions involved in cell movement such as invadopodia and lamellipodia. Scale bar = 100 µm or 20 µm .

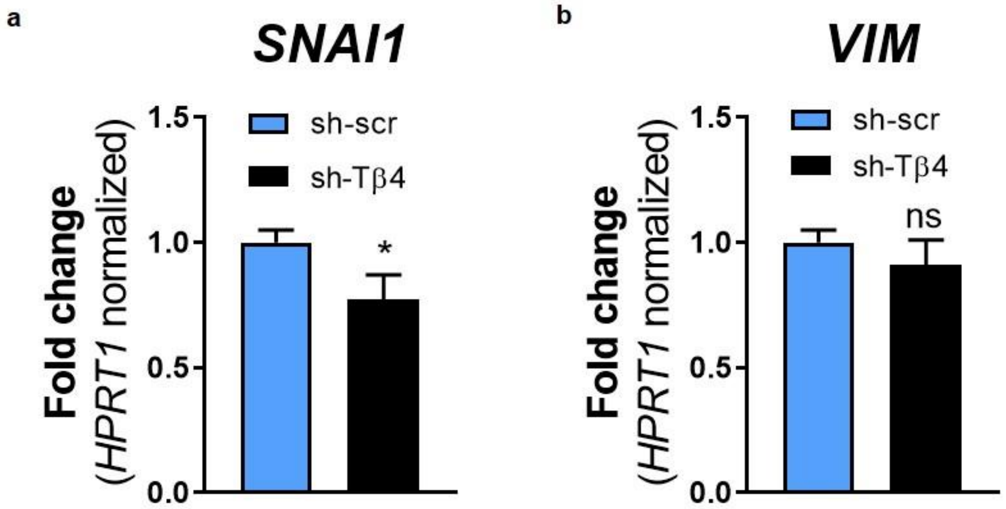

Supplementary Figure 15. qRT-PCR analysis of *SNAI1* (a) *VIM* (b) expression level in A375-*shscr* and A375-*shTMSB4X* clones.

1.2 Supplementary Tables

Antibody	Company	Immunocyt. /PLA	WB
rabbit anti-Tβ4 (FL-44)	Santa Cruz Biotechnology Inc.	1:50	-
rabbit anti-cortactin (H-191)	Santa Cruz Biotechnology Inc.	1:50	1:200
rabbit anti-Tks-5 (M-300)	Santa Cruz Biotechnology Inc.	1:50	1:200
goat anti-HA (Y-11)	Santa Cruz Biotechnology Inc.	1:50	1:200
rabbit anti-FAK (C-903)	Santa Cruz Biotechnology Inc.	1:50	1:200
mouse anti-αVβ3	Merck-Millipore	1:100	-
rabbit anti-Tβ4	Merck-Millipore	1:100	1:1000
rabbit anti-pan laminin	Sigma-Aldrich	1:60	-
rabbit anti-ILK	Sigma-Aldrich	1:100	1:1000
rabbit anti-total actin C11	Sigma-Aldrich	1:100	-
mouse anti-total actin AC40	Sigma-Aldrich	1:100	-
rabbit anti-α-parvin	Cell Signaling	1:400	-
rabbit anti-vimentin (D21H3)	Cell Signaling	-	1:1000
rabbit anti-b-catenin (D10A8)	Cell Signaling	-	1:1000
rabbit anti-SNAI2 (C19G7)	Cell Signaling	-	1:1000
rabbit anti-SNAI1 (C15D3)	Cell Signaling	-	1:1000
rabbit anti-ZO-1 (D7D12)	Cell Signaling	-	1:1000
rabbit anti-ZEB1 (D80D3)	Cell Signaling	-	1:1000
mouse anti-ZO-1 (1A12)	Thermo Fisher Scientific	1:200	-
mouse anti-vinculin (V284)	AbD Serotec (Bio-Rad)	1:100	1:1000
HRP-conjugated anti-rabbit	Cell Signaling	-	1:4000
HRP-conjugated anti-mouse	Cell Signaling	-	1:4000
donkey anti-goat-Alexa Fluor® 488	Invitrogen	1:200	-
donkey anti-mouse-Alexa Fluor® 488	Invitrogen	1:200	-
donkey anti-mouse-Alexa Fluor® 568	Invitrogen	1:200	-
donkey anti-mouse-Alexa Fluor® 647	Invitrogen	1:200	-
donkey anti-rabbit-Alexa Fluor® 488	Invitrogen	1:200	-
donkey anti-rabbit-Alexa Fluor® 568	Invitrogen	1:200	-

Supplementary Table 1. List of antibodies used in the study.

Primer	Sequence	Amplicon size (nt)	Tm (°C)
*TMSB4X*_f<br>*TMSB4X*_r	5'→caaccatgtctgacaaacc<br>5'→aaggcagtggatttttctct	96	60<br>60
*TMSB10*_f<br>*TMSB10*_r	5'→ccagacatgggggaaat<br>5'→caatggtctctttggtcg	94	60<br>60
*TMSB15*_f<br>*TMSB15*_r	5'→atgagtgataagccagacttg<br>5'→cactctttctcttgctggatag	122	60<br>60
*VIM*_f<br>*VIM*_r	5'→aaagtccgcacattcgagca<br>5'→ggtggacgtagtcacgtagc	482	60<br>60
*SNAI*1_f<br>*SNAI*1_r	5'→accccaatcggaagcctaac<br>5'→tcccagatgagcattggcag	151	60<br>60
*HPRT1*_f<br>*HPRT1*_r	5'→gaccagtcaacaggggacat<br>5'→gcttgcgaccttgaccatct	165	60<br>60

Supplementary Table 2. List of qPCR primers used in the study.

Vector	Primers sequences	Description
pLVX-IRES-puro	5'→tagaggatctatttccggtgaattcaccatgtctgacaaaccc<br>5'→taggggggggggggagggagaggggcgggatccttacgattcgcctgcttg<br>5'→aaggggctgaaggatgcccagaaggtaccccattgtatgggatctgatctgg<br>5'→aggcgcaccgtgggcttgtactcggtcatattatcatcgtgtttttcaaag<br>5'→tttgaaaaacacgatgataatatgaccgagtacaagcccacggtgcgc<br>5'→agaggttgattgttccagacgcgttcaggcaccgggcttgcgggtcatg<br>5'→atgacccgcaagcccggtgcctgaacgcgtctggaacaatcaacctc<br>5'→tgccttgtaagtcattggtcttaaaggtacctgaggtgtgactggaaaacccac	From pLVX-IRES-tdTomato-FlagAkt1 plasmid, a gift from Eva Gonzalez (Addgene plasmid # 64831) (Kajno et al., 2015).there were removed sequnces encoding FlagAkt1, tdTomato, part of IRES and WPRE sequences by *EcoR*I and *Kpn*I restriction enzymes. Next we reconstructed IRES and WPRE sequences and simultaneously cloned cDNA encoding puromycin resistance gene amplified from the SHC016 vector (Sigma-Aldrich).
pLVX-IRES-puro-3xHA-Tβ4	5'→tagaggatctatttccggtgaattcaccatggattacccatacg<br>5'→cactcatggtgaattcagaagcgtaatctg<br>5'→ttctgaattcaccatgagtgataagccag<br>5'→taggggggggggggagggagaggggcgggatccttatgatgtttgaacacactctttc	As a backbone vector was used **pLVX-IRES-puro**, which generation is described above. Between *EcoR*I and *BamH*I restriction sites there were cloned sequences coding 3xHA tag amplified from p3xHA-C1 plasmid (Müller et al., 2012) and cDNA encoding Tβ4 amplified from cDNA of the A375 cells.
pLVX-IRES-puro-3xHA-Tβ10	5'→tagaggatctatttccggtgaattcaccatggattacccatacg<br>5'→cagacatggtgaattcagaagcgtaatctg<br>5'→ttctgaattcaccatgtctgacaaaccc<br>5'→taggggggggggggagggagaggggcgggatccttacgattcgcctgcttg	As a backbone vector was used **pLVX-IRES-puro**, which generation is described above. Between *EcoR*I and *BamH*I restriction sites there were cloned sequences encoding 3xHA tag amplified from p3xHA-C1 plasmid (Müller et al., 2012) and cDNA encoding Tβ10 amplified from cDNA of the A375 cells.
pLVX-IRES-puro-3xHA-Tβ15	5'→tagaggatctatttccggtgaattcaccatggattacccatacg<br>5'→ctgccatggtgaattcagaagcgtaatctg<br>5'→ttctgaattcaccatggcagacaaaccag<br>5'→taggggggggggggagggagaggggcgggatccttaggaaatttcactccgc	As a backbone vector was used **pLVX-IRES-puro**, which generation is described above. Between *EcoR*I and *BamH*I restriction sites there were cloned sequences encoding 3xHA tag amplified from p3xHA-C1 plasmid (Müller et al., 2012) and cDNA encoding Tβ15 amplified from cDNA of the A375 cells.

Supplementary Table 3. List of clonning vectors used in the study.

Number	Sequence
**TMSB4X MISSION shRNA**	
**TRCN0000158860**	ccgggagaaattcgataagtcgaaactcgagtttcgacttatcgaatttctctttttg
**TRCN0000219784**	ccggatgtctgacaaacccgatatgctcgagcatatcgggtttgtcagacatttttg
**TRCN0000163494**	ccgggaagacagagacgcaagagaactcgagttctcttgcgtctctgtcttctttttg
**TRCN0000164355**	ccggcgattgaacaggagaagcaagctcgagcttgcttctcctgttcaatcgtttttg
**TRCN0000219785**	ccggacaagcattgccttcttatttctcgagaaataagaaggcaatgcttgtttttg
**Non-Target shRNA**	
**SHC016**	ccgggcgcgatagcgctaataatttctcgagaaattattagcgctatcgcgcttttt

Supplementary Table 4. List of silencing sequence used in the study.
